# Multi-cohort machine learning identifies a ferroptosis-linked prognostic signature in lung adenocarcinoma

**DOI:** 10.3389/fbinf.2026.1921468

**Published:** 2026-09-17

**Authors:** Rana Salihoglu

**Affiliations:** 1 Department of Computer Engineering, Recep Tayyip Erdogan University, Rize, Türkiye; 2 Chair of Bioinformatics, Julius-Maximilians-Universität Würzburg, Würzburg, Germany

**Keywords:** external validation, ferroptosis, lung adenocarcinoma, machine learning, survival learning, transcriptomic signature

## Abstract

**Introduction:**

Lung adenocarcinoma (LUAD) shows marked outcome heterogeneity within clinicopathological stage groups. This study developed and externally validated a ferroptosis-linked transcriptomic risk model using a leakage-controlled multi- cohort survival-learning framework and characterized its immune context.

**Methods:**

Candidate genes were defined by combining FerrDb V3-annotated genes within a ferroptosis-associated weighted gene co-expression network analysis (WGCNA) module with a filtered WGCNA discovery branch derived from the independent GSE81089 cohort. Model development used TCGA-LUAD, GSE31210, GSE72094, and GSE136961 (1,149 patients; 344 overall-survival events) with bagged cross-cohort Cox screening, leave-one-cohort-out (LOCO) stability locking, and benchmarking of 150 survival-learning configurations. External evaluation used the GSE50081, GSE68465, and GSE30219 cohorts (912 patients; 507 events).

**Results:**

The development-selected extra survival trees configuration reached a mean leave-one-cohort-out Uno’s C-index of 0.723. The highest cohort-specific C-indices among the prespecified candidate configurations were 0.611, 0.691, and 0.699 and arose from different model configurations. Applying the same development-selected configuration to all three external cohorts yielded Harrell’s C-indices of 0.605, 0.675, and 0.682 and 5-year Uno’s C-indices of 0.605, 0.678, and 0.696. In TCGA-LUAD, the stored out-of-fold molecular score remained prognostic after TNM stage adjustment (hazard ratio per standard deviation 2.32, 95% confidence interval: 1.39−3.87; p = 0.0013).

**Discussion:**

At 3 years and 5 years, the combined TNM-plus-score models showed close calibration and modest gains in discrimination, while decision-curve analysis identified positive incremental net benefit only over restricted threshold ranges. Low-risk tumors were enriched for interferon, complement, and immune-cell programs. These computational findings require further prospective assay-level and experimental validation before clinical implementation.

## 1 Introduction

In 2022, approximately 2.5 million new cases of lung cancer were diagnosed, and the disease caused 1.8 million deaths globally, remaining the leading cause of cancer mortality (Bray et al., 2024). Lung adenocarcinoma (LUAD) is the dominant histological subtype, and outcome heterogeneity persists even among patients with similar stages and treatment contexts. Current clinicopathologic staging captures tumor extent but does not fully resolve transcriptomic, immune, and cell-death programs that shape survival. Consequently, molecular signatures that are reproducible across independent cohorts and platforms may improve biological risk stratification, if feature selection is leakage-controlled and externally validated.

Ferroptosis is an iron-dependent regulated cell-death process driven by phospholipid peroxidation and redox failure. It was originally distinguished from apoptosis, necrosis, and autophagy by its iron dependency and sensitivity to lipid reactive oxygen species (Dixon et al., 2012). Studies established GPX4 as a central suppressor of ferroptotic death by detoxifying lipid hydroperoxides (Friedmann Angeli et al., 2014; Yang et al., 2014), while the FSP1-CoQ10 axis later defined a GPX4-independent ferroptosis-resistance system (Doll et al., 2019). A coherent integrative framework defines ferroptosis as a redox/metabolism-coupled regulated cell-death program intertwined with iron, lipid, glutathione, and selenium biology (Ito et al., 2024; Jiang et al., 2021; Stockwell et al., 2017). These findings, together with broader reviews of ferroptosis regulation in cancer (Doll et al., 2019; Jiang et al., 2021; Yang et al., 2014), support ferroptosis as a biologically plausible LUAD vulnerability. FerrDb provides a manually curated catalog of ferroptosis regulators, markers, and disease associations, and supports systematic incorporation of ferroptosis biology into transcriptomic modeling (Zhou et al., 2026). Critically, ferroptosis is coupled to anti-tumor immunity: immunotherapy-activated CD8^+^ T cells repress SLC3A2/SLC7A11 via interferon-γ to enhance tumor-cell ferroptosis (Wang et al., 2019), and radiotherapy and immunotherapy converge on SLC7A11 repression to promote tumor lipid oxidation (Lang et al., 2019).

The relevance of ferroptosis in LUAD is not restricted to tumor-cell autonomous lipid metabolism. Activated CD8^+^ T cells can promote tumor-cell ferroptosis through interferon-γ-mediated repression of SLC3A2 and SLC7A11 (Wang et al., 2019), and radiotherapy can cooperate with immunotherapy to enhance lipid oxidation through convergent SLC7A11 suppression (Lang et al., 2019). Therefore, ferroptosis-associated transcriptional states may encode an immune-contextual phenotype. This is particularly important in LUAD, where immune-checkpoint therapy, targeted therapy, and conventional treatment responses are influenced by tumor microenvironmental state.

Several ferroptosis-related prognostic signatures have been proposed for LUAD (Ren et al., 2021; Zhang Z. et al., 2021; Zhu et al., 2021), and ferroptosis gene sets have been connected with immune infiltration, mutation patterns, drug-response surrogates, and clinical risk (Ma et al., 2021; Sun et al., 2022; Wang et al., 2021). However, many published models remain vulnerable to optimistic performance estimates because feature selection is often performed in a single discovery cohort, external validation is limited, and cross-platform transportability or feature-selection stability is incompletely addressed. For a clinically interpretable prognostic model, ferroptosis-linked genes should therefore be selected not only for marginal survival association but also for reproducibility across heterogeneous cohorts and biological anchoring within a ferroptosis-relevant network structure (Ito et al., 2024; Jiang et al., 2021).

Weighted gene co-expression network analysis (WGCNA) can reduce the supervised-learning search space by identifying co-regulated gene modules associated with biological traits, thereby constraining downstream modeling to biologically coherent candidates (Langfelder and Horvath, 2008). Recent integrative biomarker-discovery studies further support combining WGCNA, machine learning (ML) feature selection, pathway annotation, and survival modeling to prioritize prognostic cancer subnetworks from heterogeneous transcriptomic cohorts (Li et al., 2025; Salihoglu et al., 2025). Here, survival learning denotes supervised modeling of censored time-to-event outcomes. Building on this rationale, we developed a leakage-controlled multi-cohort ML framework that integrates FerrDb annotation, an independent WGCNA-derived ferroptosis module, bagged cross-cohort Cox screening, and leave-one-cohort-out (LOCO) stability locking. The resulting 25-gene ferroptosis-linked transcriptomic risk model was trained in four LUAD cohorts. It was evaluated without further tuning in three independent external cohorts and characterized for stage-adjusted prognostic value, calibration, and immune-contextual biology. Additional post-locking analyses evaluated clinical utility, proportional-hazards-robust sensitivity, nomograms, calibration, decision curves, and signature stability.

## 2 Materials and methods

The analytic pipeline is organized in three parts: (i) signature discovery and locking, comprising WGCNA module identification on an independent cohort, cohort-pooled LOCO training with bagged meta-univariate selection and stability locking; (ii) external validation and clinical-epidemiologic inference, comprising stage-stratified Cox analysis, multi-cohort meta-analysis, proportional-hazards diagnostics, fixed-horizon calibration, decision-curve analysis and nomogram construction; and (iii) biological characterization, comprising pathway enrichment, protein–protein interaction (PPI) network analysis and microenvironment deconvolution. Detailed surrogate-SHAP/permutation interpretation, immunotherapy-response propensity, signature-reversal connectivity, target druggability, and structural prioritization were treated as supportive analyses and are reported in the Supplementary Material. Post-locking clinical-utility, robustness, and comparative analyses used stored outputs without feeding back into feature selection, hyperparameter tuning, or external predictions (Figure 1). Model terminology and abbreviations are summarized in Supplementary Tables S1, S2.

**FIGURE 1:**
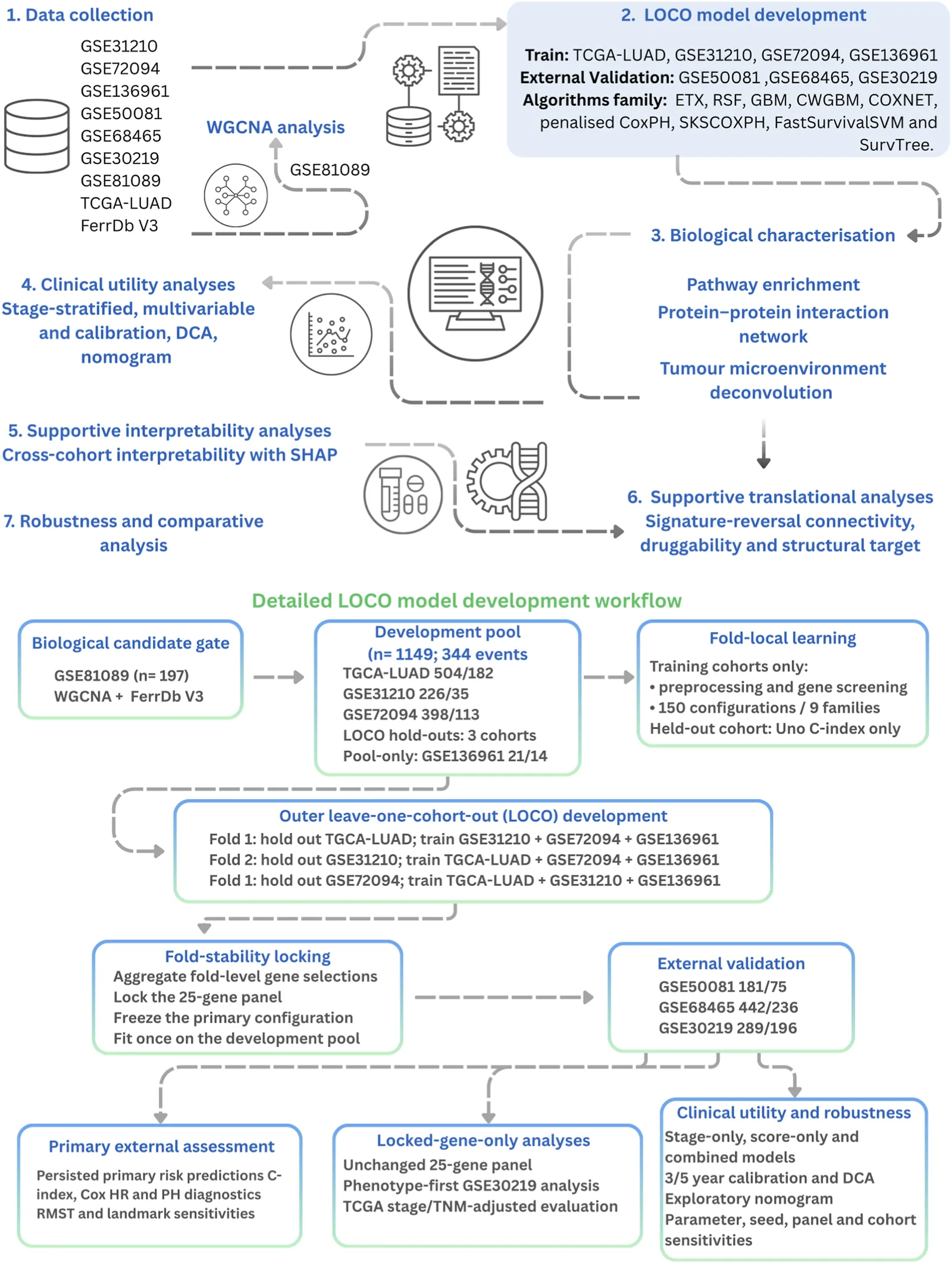
Leakage-controlled multi-cohort survival-learning workflow. Candidate-gene definition used the independent GSE81089 WGCNA/FerrDb gate. In each outer leave-one-cohort-out (LOCO) fold, preprocessing, gene selection, and hyperparameter tuning were restricted to training cohorts, and performance was estimated only in the held-out development cohort. Fold-level selections were aggregated to lock the 25-gene panel and to identify a development-selected reference configuration before reserved external evaluation. External cohorts did not contribute to feature selection, tuning, or cut-point optimization.

### 2.1 Datasets, cohort curation, and harmonization

TCGA-LUAD RNA-seq and clinical data were obtained from the NCI Genomic Data Commons (GDC), which provides uniformly processed and harmonized cancer genomic data (Zhang A. et al., 2021; Salihoglu et al., 2024), and were interpreted in the context of the TCGA-LUAD molecular-profiling resource. The Gene Expression Omnibus (GEO) cohorts included GSE31210, GSE72094, GSE136961, GSE50081, GSE68465, GSE30219, and GSE81089.

The machine learning model was based on the TCGA-LUAD GSE31210, GSE72094, and GSE136961 cohorts (Table 1), while the GSE50081, GSE68465, and GSE30219 cohorts were kept for external validation. GSE81089 was used exclusively for WGCNA-based candidate-gene prioritization (Langfelder and Horvath, 2008) and was not used for model fitting, thresholding, or performance estimation. Expression matrices were log_2_-transformed when appropriate, mapped to HUGO Gene Nomenclature Committee (HGNC) symbols, collapsed to genes by maximum expression, and filtered to remove ambiguous probes. Stage was harmonized into ordered categories (I–IV) with an explicit missing-stage indicator. Follow-up time was unified to days; overall survival (OS) was the only event endpoint. Cohort assignment to training, external evaluation, and WGCNA discovery was fixed *a priori*, and external cohorts were untouched until final evaluation. Cohort-aware z-standardization was used, and normalization parameters estimated on training data were transferred to held-out cohorts to avoid information leakage.

**TABLE 1:** Cohort inventory and quality control summary.

Cohort	Role	n	Events	Event rate	Median OS (days)	Platform	Reference
TCGA-LUAD	Training (pool)	504	182	0.36	657.5	RNA-seq (GDC; STAR, TPM)	The Cancer Genome Atlas Research Network (2014)
GSE31210	Training (pool)	226	35	0.16	1,744.5	Affymetrix HG-U133 plus 2.0	Okayama et al. (2012)
GSE72094	Training (pool)	398	113	0.28	824	Rosetta/Merck Affymetrix	Schabath et al. (2016)
GSE136961	Training (pool, small)	21	14	0.67	260	Affymetrix (small LUAD subset)	Hwang et al. (2020)
GSE50081	External validation	181	75	0.41	1,503	Affymetrix HG-U133 plus 2.0	Der et al. (2014)
GSE68465	External validation	442	236	0.53	1,430.6	Affymetrix HG-U133A	Shedden et al. (2008)
GSE30219	External validation	289	196	0.68	1,613.2	Affymetrix HG-U133 plus 2.0	Rousseaux et al. (2013)
GSE81089	WGCNA analysis	197	—	—	—	Illumina HiSeq (NSCLC)	Djureinovic et al. (2016)

In addition to RNA features, we computed cohort-standardized auxiliary descriptors representing aggregate ferroptosis-driver expression, ferroptosis-suppressor expression (FerrDb V3; Zhou et al., 2026), WGCNA module activity, and derived interaction terms. Feature ranking, standardization, thresholding, and calibration were performed within training data only to prevent external-validation leakage.

### 2.2 Candidate-gene definition

Candidates were derived from an independent WGCNA analysis (Langfelder and Horvath, 2008) in GSE81089, which was not used for model fitting, feature selection, or external validation. Expression data were log_2_-transformed, sample- and variance-filtered, median-imputed where required, and restricted to the top 8,000 genes by median absolute deviation. WGCNA used a signed adjacency network, signed topological overlap matrix (TOM), and biweight midcorrelation; the soft-thresholding power was the smallest candidate achieving signed scale-free topology fit *R* ^2^ ≥ 0.80 (or the maximum-R^2^ power if none met the criterion). Module detection used blockwiseModules (minimum module size = 30; merge cut-height = 0.25; deepSplit = 2).

Sample-level ferroptosis driver and suppressor scores were estimated by single-sample gene-set enrichment analysis (ssGSEA) with a rank-based fallback, using FerrDb sets (Zhou et al., 2026). The composite ferroptosis trait was calculated as driver-score minus suppressor-score. Module-trait correlations were calculated using Pearson r with the composite trait (and Spearman correlation as a sensitivity analysis); candidate modules required Pearson r ≥ 0.20 and Benjamini–Hochberg false discovery rate (FDR) ≤ 0.10. The first trait-correlated module containing at least three FerrDb genes was selected. The selected module was partitioned into a conservative branch (FerrDb driver/suppressor/marker genes within the module, ranked by hub score = |module membership (MM) | × |gene significance (GS)|) and a discovery branch (non-FerrDb genes with |MM| ≥ 0.30, |GS| ≥ 0.20, and univariable Cox *p* < 0.05). The WGCNA-derived input to downstream learning was the union of conservative and filtered discovery candidates.

### 2.3 Survival-signature learning: LOCO, bagged meta-univariate selection, and stability locking

Survival-signature learning was performed within a LOCO framework to quantify cross-cohort transportability and reduce cohort-specific overfitting. TCGA-LUAD, GSE31210, and GSE72094 served sequentially as internal hold-out cohorts, whereas GSE136961 contributed only to pooled training because of its limited sample and event count. The GSE50081, GSE68465, and GSE30219 cohorts were kept independent from feature selection, hyperparameter tuning, thresholding, and ensemble optimization.

During LOCO training, RNA features were z-scored within training cohorts, and hold-out data were standardized without outcome information. Clinical stage was encoded ordinally with a deficiency indicator. Prespecified ferroptosis/WGCNA descriptors were permitted only as auxiliary covariates and could not replace the locked RNA-gene signature.

Within each LOCO fold, RNA features were variance-filtered to a maximum of 2,400 genes and, when enabled, intersected with WGCNA-derived candidates. Candidate genes were ranked by bagged meta-univariate Cox screening across eight bootstrap resamples, using Cox proportional-hazards regression (Cox, 1972) and bootstrap resampling principles (Efron, 1992). Cohort-wise Cox statistics were combined into meta-Z scores to prioritize genes with consistent prognostic direction and discrimination. Fold-level 50-gene panels were merged by LOCO stability; genes selected in at least 50% of folds were prioritized, and the final RNA signature was capped at 25 genes. Nine survival-learning families were benchmarked across 150 hyperparameter configurations: extra survival trees (ETX), random survival forest (RSF), gradient boosting survival analysis (GBM), component-wise GBM (CWGBM), elastic net Cox (COXNET), penalized CoxPH, scikit-survival CoxPH (SKSCOXPH), fast survival SVM (FASTSVM), and survival tree (Pedregosa et al., 2011). The primary discrimination metric was Uno’s inverse-probability-of-censoring-weighted (IPCW) C-index, with Harrell’s C used as a fallback. Bootstrap confidence intervals were computed by resampling. Ensembles were built over the top-ranked algorithms by rank averaging, weighted rank aggregation, softmax weighting, Borda aggregation, trimmed rank aggregation, hybrid rank-Z scoring, out-of-fold Cox stacking, and ETX/RSF/GBM hybridization. Proportional-hazards assumptions were evaluated cohort-wise using scaled Schoenfeld residuals, where violations were detected.

The locked gene set includes the 25 genes aggregated from fold-level selection frequencies. The development-selected reference configuration denotes the ETX model ranked first on the development-only LOCO ranking. Per-cohort best single-model external results were retained as a descriptive candidate-model summary, whereas the primary fixed-configuration external evaluation applied the same development-selected reference configuration unchanged to all three external cohorts. The locked panel, reference model, stacked model, out-of-fold predictions, and refitted clinical models were treated as distinct analytical objects. Detailed procedures for the fixed-configuration external evaluation are provided in Supplementary Material S1.1.

### 2.4 Clinical-inference, external validation, and calibration analyses

In TCGA-LUAD, an exploratory, outcome-informed clinical-inference analysis used the ML-derived full-training prognostic candidate panel rather than directly refitting the locked 25-gene external-validation signature. Genes were harmonized to TCGA-LUAD patient-level expression and evaluated with both univariable and stage-adjusted Cox models. Per-gene TCGA-adjusted Cox coefficients were combined with cross-cohort meta-univariate evidence, including the cross-cohort meta-analysis z-statistic (z_meta_) and bagged-selection fraction, into an evidence-weighted coefficient. Genes with finite stage-adjusted coefficients and adjusted *p*-value <0.20 were retained in the TCGA-specific weighted risk model, with a fallback to the highest-ranked genes if fewer than five passed. The resulting TCGA-refitted weighted gene set was used to compute a normalized linear risk score from z-scored expression, with sign carried by the per-gene coefficients.

Continuous and median-dichotomized TCGA risk scores were analyzed by overall and stage-stratified Cox modeling (stage I, II, III, and IV; early I–II vs. late III–IV). Multivariable Cox models compared risk-only, clinicopathologic-only, and risk + clinicopathologic models, with clinicopathologic covariates selected from age, sex, stage group, T-stage, N-stage, and M-stage after missingness and variability checks. A stage × risk interaction was also tested. Time-dependent areas under the receiver operating characteristic curve (AUCs) at 1 year, 3 years, and 5 years, bootstrap optimism correction, PH diagnostics, calibration curves, decision-curve analysis, and a nomogram were generated using the prespecified survival-analysis packages. For external validation, the continuous risk score and median-dichotomized groups were refitted by Cox regression in each external cohort (GSE50081, GSE68465, and GSE30219). Pooled effects were estimated using random-effects meta-analysis on log hazard ratio (log-HR) estimates with DerSimonian–Laird τ^2^, reporting pooled HR, 95% CI, *p*-value, I^2^, and τ^2^. LOCO sensitivity analysis quantified each cohort’s contribution.

In an additional post-locking analysis, out-of-fold predictions from the selected extra survival trees configuration were linked to TCGA-LUAD patients using the original sample order and exact survival matching. Relative-hazard analyses compared categorical American Joint Committee on Cancer/tumor-node-metastasis (AJCC/TNM) stage, the standardized molecular score, and their combination in a common stage-complete cohort. Cox models used Efron partial likelihood, likelihood-ratio comparisons, and Schoenfeld-residual diagnostics; a stage-by-score interaction was also tested. Because proportional hazards were not uniformly supported for stage, absolute-risk analyses were performed separately at 3 years and 5 years. Deaths observed by each horizon were treated as cases, patients surviving beyond the horizon as controls, and patients censored before the horizon were excluded from the corresponding binary outcome. Inverse-probability-of-censoring weights were estimated from the Kaplan–Meier censoring distribution. IPCW-weighted ridge logistic models were fitted for TNM stage alone, the molecular score alone, and their combination, with regularization selected by cross-validation and non-negative coefficients imposed for ordered stage and the oriented score. Time-dependent AUC, IPCW Brier score, calibration intercept and slope, expected-to-observed ratio, continuous net reclassification improvement (NRI), and integrated discrimination improvement (IDI) were derived from the same horizon-specific predictions. Decision-curve analysis compared TNM, molecular-score, and combined models with treat-all and treat-none strategies across threshold probabilities from 0.02 to 0.60. Three- and 5-year nomograms mapped the coefficients of the combined fixed-horizon models to total points and overall-survival probability. Calibration, decision curves, discrimination, and incremental metrics were internally validated using 1,000 bootstrap resamples with optimism correction. Restricted mean survival time at 3 years and 5 years and conditional landmark analyses at 12 months, 24 months, and 36 months complemented Cox inference. Detailed specifications are provided in Supplementary Material S1.2.

### 2.5 Biological pathway, network, and tumor-microenvironment analyses

Biological characterization of the ferroptosis-linked signature used pathway enrichment, PPI analysis, and tumor-microenvironment (TME) deconvolution. Over-representation analysis (ORA) and gene set enrichment analysis (GSEA) were applied to the 50-gene extended signature using clusterProfiler/ReactomePA-compatible workflows (Yu et al., 2012; Yu and He, 2016) and MSigDB-derived gene sets (Subramanian et al., 2005). Enrichment was stratified by risk-promoting and protective genes, with Benjamini–Hochberg correction applied unless otherwise specified (Benjamini and Hochberg, 1995).

PPI analysis used STRING v12.0 *Homo sapiens* interactions at medium confidence (combined score ≥0.40) (Szklarczyk et al., 2023; Salihoglu et al., 2023). The largest connected component was analyzed with graph-theoretic centrality metrics; hub genes were defined by consensus centrality; and Louvain clustering identified network communities (Blondel et al., 2024). Multiple TME deconvolution and estimation outputs were generated per sample across six cohorts using the immuno-oncology biological research (IOBR) framework (Zeng et al., 2021): ESTIMATE (ImmuneScore, StromalScore, ESTIMATEScore, TumorPurity), xCell, MCPcounter, and quanTIseq, with CIBERSORT-style outputs recorded where available (Aran et al., 2017; Finotello et al., 2019; Yoshihara et al., 2013). For each feature, cohort-wise effect sizes (rank-biserial r and Wilcoxon p for high-vs. low-risk) and random-effects meta-estimates (Stouffer Z, Benjamini–Hochberg FDR) were computed. A gene set variation analysis (GSVA) signature of package-derived immune programs was scored and survival-meta-analyzed across cohorts.

### 2.6 Supportive interpretability and translational analyses

Cross-cohort interpretability, latent immunotherapy-response propensity, signature-reversal connectivity, druggability mapping, and structural prioritization were retained as supportive, hypothesis-generating analyses (Supplementary Material S1.3). Surrogate extra trees models and SHAP/permutation rankings were used only to approximate stored risk scores and were not used for feature selection or performance estimation (Geurts et al., 2006; Li, 2022; Graf et al., 1999). Immune-context scoring used FerrDb and MSigDB collections (Zhou et al., 2026; Subramanian et al., 2005), while druggability and structural annotations used DGIdb, ChEMBL, Open Targets, UniProt, PDB, and AlphaFold (Freshour et al., 2021; Zdrazil et al., 2024; Ochoa et al., 2023; Consortium, 2015; Varadi et al., 2022). These analyses were interpreted as model-explanation and prioritization outputs.

### 2.7 Signature stability and comparative analyses

Post-locking sensitivity analyses varied the minimum LOCO selection frequency (0.33, 0.50, 0.67, and 1.00), signature-size cap (20 genes, 25 genes, and 30 genes), directional-consistency requirements, and the minimum number of contributing development cohorts. Development-cohort jackknife analyses and exclusion of the small GSE136961 cohort evaluated sensitivity to cohort composition, with absent platform genes recorded. Algorithm-family robustness was summarized across all 150 stored configurations from nine survival-learning families. Published LUAD ferroptosis-related signatures were compared descriptively by signature size, discovery strategy, model class, development and validation cohorts, leakage control, experimental validation, and reported discrimination. No head-to-head performance ranking was undertaken because the studies used different endpoints, horizons, cohorts, and metrics. Full procedures are provided in Supplementary Material S1.4.

## 3 Results

### 3.1 Cohort assembly, gene pool, and 25-gene locked signature

After harmonization and quality control (Table 1), the training pool comprised 1,149 LUAD patients with 344 events across the TCGA-LUAD, GSE31210, GSE72094, and GSE136961 cohorts (with event rates ranging from 15.5% to 66.7%). The external-evaluation set comprised 912 patients with 507 events from the GSE50081, GSE68465, and GSE30219 cohorts. Event-rate heterogeneity was handled during training using inverse cohort-size × event-rate sample weighting.

In the independent GSE81089 cohort, the selected soft-thresholding power achieved the predefined scale-free topology criterion while preserving network connectivity. The MEgreen module showed the strongest and statistically robust association with FERRO_TRAIT (Pearson r = 0.595, FDR = 3.7 × 10–19) and with FERRO_DRIVER (r = 0.592). Within MEgreen, 109 FerrDb genes formed the conservative candidate pool. An additional 30 genes were retained from the discovery branch, giving a 139-gene WGCNA-derived input set (Figure 2).

**FIGURE 2:**
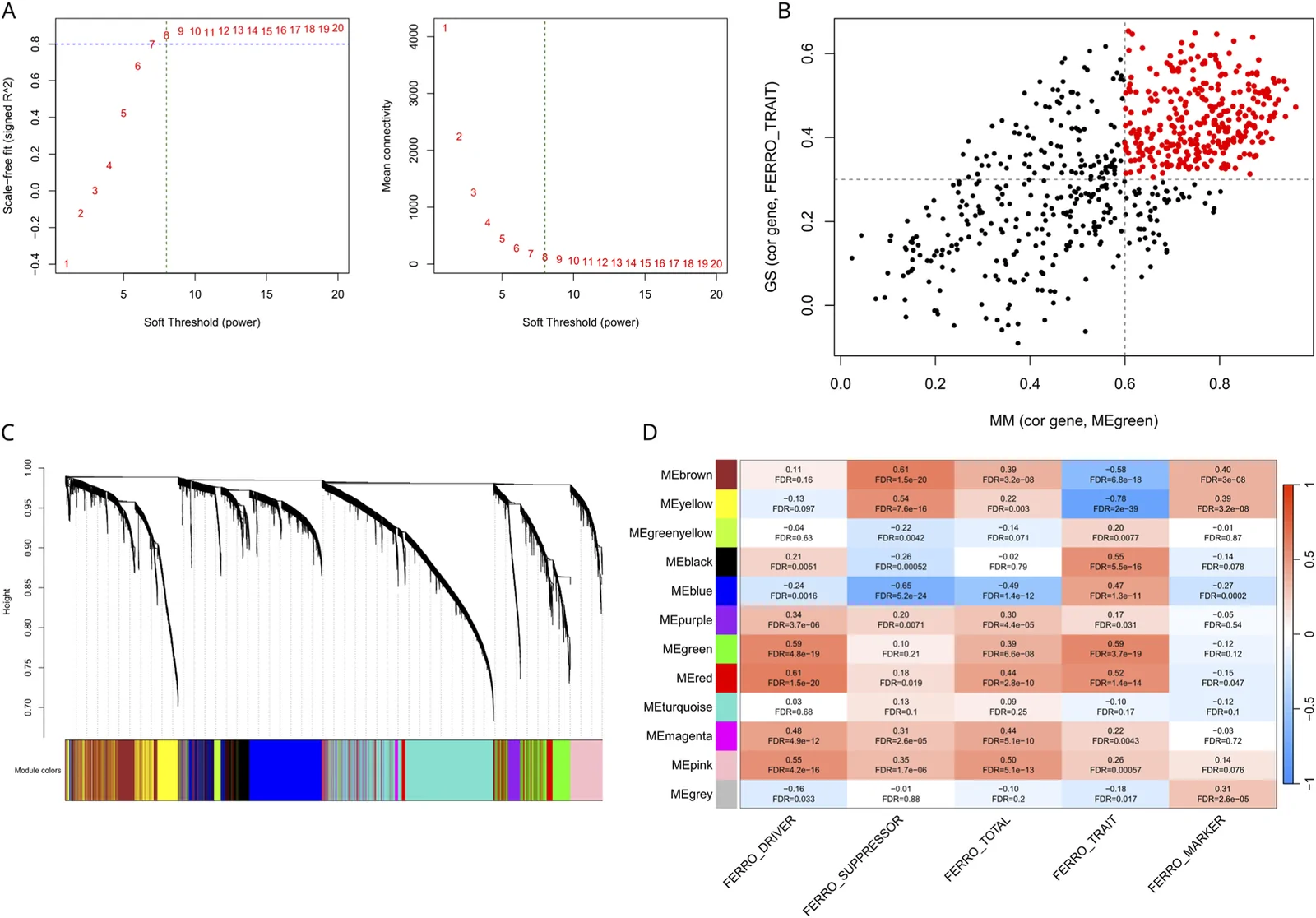
WGCNA identification of a ferroptosis-associated module. **(A)** Scale-free topology fit and mean connectivity across soft-thresholding powers. **(B)** Module membership versus “FERRO_TRAIT” gene significance in the selected module. **(C)** Gene dendrogram and dynamic module assignment. **(D)** Module-trait correlation heatmap identifying the ferroptosis-associated green module.

Bagged cross-cohort meta-univariate selection retained 50 top-ranked genes with full selection frequency across the eight bootstrap bags. LOCO stability locking yielded 25 genes, and 22 of those were members of both FerrDb and the MEgreen module, with three genes (LHFPL2, ALOX5AP, and ITM2A) from WGCNA-only co-passengers (Table 2).

**TABLE 2:** Locked 25-gene signature with direction, source, bagged-selection frequency, and TCGA-refitted per-gene Cox statistics adjusted for stage. Zmeta is the cross-cohort meta-univariate z-statistic obtained from the development cohorts; bag_frac_ is the fraction of the eight bootstrap bags in which the gene was selected; β_adj_ and HR (95% CI) are stage-adjusted Cox estimates in TCGA-LUAD; p is the stage-adjusted Cox p-value for that estimate.

Gene	Direction	Source	z_meta_	bagf_rac_	β_adj_	HR (95% CI)	*p*
VNN1	Risk	FerrDb + WGCNA	+4.14	1.00	+0.197	1.22 (1.06–1.40)	0.005
RIPK2	Risk	FerrDb + WGCNA	+3.03	1.00	+0.318	1.37 (1.14–1.66)	0.001
B4GALT1	Risk	FerrDb + WGCNA	+2.62	1.00	+0.350	1.42 (1.17–1.73)	4.35 × 10^−4^
DNAJA1	Risk	FerrDb + WGCNA	+1.88	1.00	+0.278	1.32 (1.07–1.64)	0.011
LHFPL2	Risk	WGCNA	+1.45	1.00	+0.203	1.22 (0.96–1.57)	0.110
CXCL10	Risk	FerrDb + WGCNA	+0.96	1.00	+0.010	1.01 (0.93–1.10)	0.811
CD274	Risk	FerrDb + WGCNA	+0.47	1.00	+0.031	1.03 (0.91–1.17)	0.623
FASLG	Protective	FerrDb + WGCNA	−1.80	1.00	−0.082	0.92 (0.74–1.14)	0.455
CCR5	Protective	FerrDb + WGCNA	−1.89	1.00	−0.078	0.93 (0.81–1.06)	0.257
CTLA4	Protective	FerrDb + WGCNA	−2.02	1.00	−0.124	0.88 (0.75–1.04)	0.129
PPM1K	Protective	FerrDb + WGCNA	−2.10	1.00	−0.135	0.87 (0.71–1.08)	0.206
ALOX5AP	Protective	WGCNA	−2.14	1.00	−0.077	0.93 (0.81–1.06)	0.276
FAS	Protective	FerrDb + WGCNA	−2.31	1.00	−0.026	0.97 (0.83–1.14)	0.745
CD28	Protective	FerrDb + WGCNA	−2.37	1.00	−0.255	0.77 (0.58–1.03)	0.076
NCKAP1L	Protective	FerrDb + WGCNA	−2.37	1.00	−0.097	0.91 (0.78–1.06)	0.217
PRKCB	Protective	FerrDb + WGCNA	−2.56	1.00	−0.643	0.53 (0.27–1.03)	0.059
RASSF2	Protective	FerrDb + WGCNA	−2.62	1.00	−0.125	0.88 (0.74–1.05)	0.166
PTPRC	Protective	FerrDb + WGCNA	−2.60	1.00	−0.164	0.85 (0.72–0.99)	0.042
MEF2C	Protective	FerrDb + WGCNA	−2.72	1.00	−0.138	0.87 (0.64–1.18)	0.368
OGT	Protective	FerrDb + WGCNA	−2.87	1.00	−0.158	0.85 (0.72–1.01)	0.067
FYN	Protective	FerrDb + WGCNA	−3.04	1.00	−0.190	0.83 (0.61–1.12)	0.214
IL7R	Protective	FerrDb + WGCNA	−3.09	1.00	−0.105	0.90 (0.80–1.02)	0.093
MAL	Protective	FerrDb + WGCNA	−3.09	1.00	−0.273	0.76 (0.61–0.95)	0.017
ITM2A	Protective	WGCNA	−3.16	1.00	−0.048	0.95 (0.83–1.09)	0.489
CCR7	Protective	FerrDb + WGCNA	−3.25	1.00	−0.132	0.88 (0.75–1.02)	0.088

Directionality was assigned by the sign of the cross-cohort meta-univariate z-statistic (z_meta_), which was the criterion used to build the signature. The strongest cross-cohort protective signals were observed for CCR7 (z_meta_ = −3.25), ITM2A (−3.16), MAL and IL7R (both −3.09), FYN (−3.04), and OGT (−2.87). The strongest risk-promoting signals were observed for VNN1 (+4.14), RIPK2 (+3.03), and B4GALT1 (+2.62). Seven of the 25 genes had positive z_meta_ values (VNN1, RIPK2, B4GALT1, DNAJA1, LHFPL2, CXCL10, and CD274). The remaining 18 genes were protective, and thus the signature was asymmetric, with a protective arm dominated by adaptive-immune-receptor biology that outweighed the risk arm both numerically and in summed |z_meta_|.

After stage-adjusted refitting in TCGA and FDR correction across the 50-gene extended panel, three risk-promoting genes retained FDR-significant associations (B4GALT1: HR 1.42, 95% CI: 1.17–1.73; FDR = 0.022; RIPK2: HR 1.37, 1.14–1.66; FDR = 0.027; VNN1: HR 1.22, 1.06–1.40; FDR = 0.050). No protective gene survived FDR correction in TCGA alone, although several were nominally significant (MAL HR 0.76, *p* = 0.017; PTPRC HR 0.85, *p* = 0.042). Cross-cohort meta-Z magnitudes varied across the locked genes and were not subject to a uniform |zmeta| threshold; panel locking was driven by fold-level selection frequency. Immune-receptor genes (CD28, CTLA4, CCR5, CCR7, IL7R, CD274, FAS, and FASLG) formed a coherent protective block whose individual TCGA effects are modest but whose additive contribution to the linear-predictor risk score is substantial.

### 3.2 Internal LOCO performance and external discrimination

Across 150 configurations spanning nine survival-learning families, extra survival trees performed best on the internal LOCO leaderboard. The best configuration, comprising 400 trees, minimum leaf size of 20, maximum features of 0.65, and a maximum depth of 8, achieved a mean LOCO Uno’s C-index of 0.723 (SD 0.043). Ten of the top fifteen models were extra survival tree variants with LOCO Uno’s C-index ≥0.721, whereas elastic-net Cox and penalized Cox models achieved lower discrimination, with LOCO Uno’s C-indices of approximately 0.69–0.71 (Figure 3).

**FIGURE 3:**
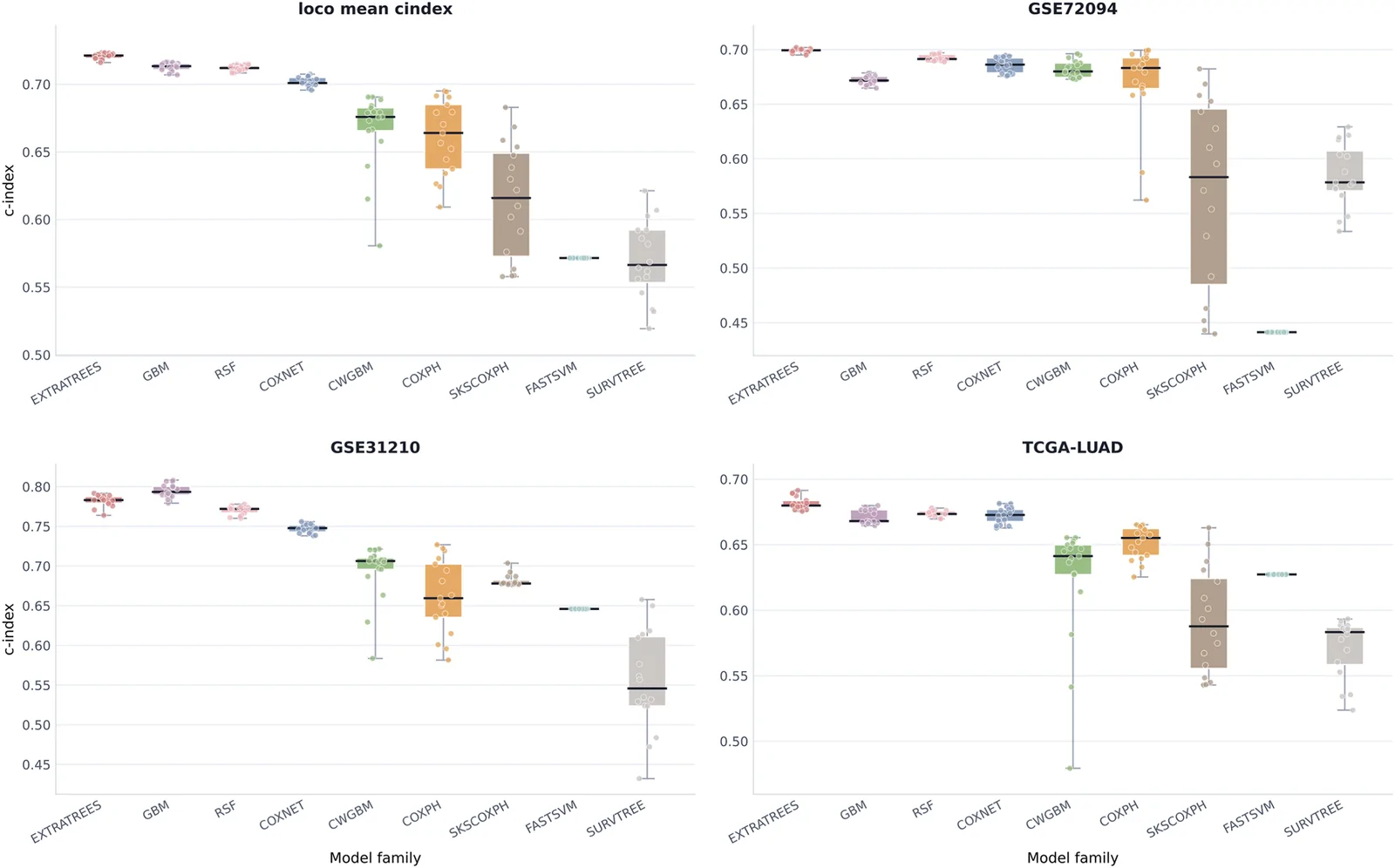
Internal LOCO benchmarking across nine survival-learning families. Extra survival trees (and related tree ensembles) achieved the strongest internal discrimination; the top configuration (400 trees; min-leaf 20; max-features 0.65; depth 8) reached LOCO Uno’s C-index of 0.723 (SD 0.043).

External validation showed moderate discrimination across independent cohorts (Table 3). The highest single-model external C-index among the prespecified candidate configurations was 0.611 (95% CI: 0.523–0.693) in GSE50081, 0.691 (0.656–0.731) in GSE68465, and 0.699 (0.655–0.734) in the complete GSE30219 cohort. These per-cohort maxima describe the completed candidate-model benchmark and should not be interpreted as performance of one common model selected across all external cohorts. Median C-indices across the top 30 models were 0.593, 0.677, and 0.664, respectively.

**TABLE 3:** Descriptive per-cohort best single-model external discrimination, proportional-hazards diagnostics and 3-year expected/observed calibration. The reported C-indices are the highest observed values among the prespecified candidate configurations in each external cohort (Supplementary Material S2.1; Supplementary Figure S1; Supplementary Table S3).

Cohort	Best single model	External C-index (95% CI)	PH p	PH violated?	E/O ratio (95% CI)	Best ensemble	Ensemble C-index
GSE50081	CWGBM_ne600_lr0.1	0.611 (0.523–0.693)	0.33	No	1.000 (0.80–1.25)	ETX_RSF_OPT	0.6
GSE68465	ENET_l10.25_amin0.1	0.691 (0.656–0.731)	6.8 × 10^−4^	Yes	0.997 (0.88–1.13)	OPT_WEIGHTED_RANK	0.68
GSE30219	COXPH_pen0.75	0.699 (0.655–0.734)	0.08	Borderline	1.004 (0.87–1.15)	OPT_WEIGHTED_RANK	0.69

Scaled Schoenfeld diagnostics indicated proportional-hazards violation in GSE68465, borderline evidence of violation in the GSE30219 cohort, and no evidence of violation in GSE50081. Therefore, effect-size interpretation was complemented by proportional-hazards robust analyses. In the fixed-configuration sensitivity analysis, the development-selected extra survival trees configuration yielded Harrell’s C-indices of 0.605, 0.675, and 0.682 and 5-year Uno’s C-indices of 0.605, 0.678, and 0.696 in the GSE50081, GSE68465, and GSE30219 cohorts, respectively (Supplementary Figure S1; Supplementary Table S3). LOCO estimates and complete-cohort Kaplan–Meier analyses retained the adverse-risk direction (Supplementary Figure S2).

### 3.3 Clinical association, external meta-analysis, and fixed-horizon utility

Three Cox models were compared in TCGA-LUAD (Table 4). The risk-only score was strongly prognostic (β = 0.597; HR 1.82, 95% CI: 1.39–2.37; *p* = 1.1 × 10^−5^). In the clinicopathologic-only model, late-stage disease (III–IV vs. I–II) was the dominant predictor (HR 2.47, 95% CI: 1.74–3.50; *p* = 3.7 × 10^−7^), whereas age, sex, and M-stage were not significant. In the combined model, the risk score remained independently prognostic (β = 0.612; HR 1.84, 95% CI: 1.38–2.47; *p* = 4.3 × 10^−5^), and late-stage disease remained the strongest clinical predictor (HR 2.34, 95% CI: 1.65–3.32; *p* = 2.0 × 10^−6^). The similar coefficients in the risk-only and combined models (β 0.597 vs. 0.612) indicate limited attenuation after clinical adjustment. Median-dichotomized risk was similarly associated with survival (HR 1.91, 95% CI: 1.40–2.61; *p* = 4.4 × 10^−5^). Time-dependent AUCs for the combined model at 1 year, 3 years, and 5 years were 0.708, 0.668, and 0.654, compared with 0.629, 0.617, and 0.632 for the clinicopathologic-only model. Adding the risk score improved model fit (likelihood-ratio *p* = 3.9 × 10^−5^).

**TABLE 4:** TCGA-LUAD clinicopathologic and stage-stratified Cox associations for the TCGA-refitted ML-derived ferroptosis-related prognostic gene panel. The upper rows compare risk-only, clinicopathologic-only, and combined models; the lower rows report stage-stratified continuous-risk-score effects and the stage × risk interaction test.

Model/term/subset	β	HR (95% CI)	*p*
Multivariable clinicopathologic models (n = 504, events = 182)			
Model A1: risk only			
Risk score	+0.597	1.82 (1.39–2.37)	1.08 × 10^−5^
Model A2: clinicopathologic only			
Age (per year)	+2.07 × 10^−5^	1.00 (1.00–1.00)	0.335
Sex (male vs. female)	−0.018	0.98 (0.72–1.34)	0.910
Stage group (late III–IV vs. early I–II)	+0.903	2.47 (1.74–3.50)	3.74 × 10^−7^
M-stage = M1	+0.022	1.02 (0.56–1.86)	0.943
M-stage = other	−0.229	0.80 (0.55–1.15)	0.227
Model A3: risk + clinicopathologic			
Risk score	+0.612	1.84 (1.38–2.47)	4.30 × 10^−5^
Age (per year)	+3.64 × 10^−5^	1.00 (1.00–1.00)	0.092
Sex (male vs. female)	−0.111	0.89 (0.66–1.22)	0.482
Stage group (late III–IV vs. early I–II)	+0.849	2.34 (1.65–3.32)	1.99 × 10^−6^
M-stage = M1	+0.128	1.14 (0.62–2.07)	0.676
M-stage = other	−0.134	0.87 (0.60–1.27)	0.484
Stage-stratified continuous-risk-score Cox models			
Stage I (n = 270, events = 65)	+0.33	1.39 (0.90–2.14)	0.14
Stage II (n = 120, events = 54)	+0.52	1.68 (1.04–2.70)	0.033
Stage III (n = 81, events = 45)	+0.97	2.63 (1.37–5.05)	0.004
Stage IV (n = 25, events = 16)	+0.74	2.09 (0.58–7.51)	0.26
Early (I–II) aggregate (n = 390, events = 119)	+0.46	1.58 (1.15–2.17)	4.9 × 10^−3^
Late (III–IV) aggregate (n = 106, events = 61)	+0.89	2.44 (1.40–4.27)	1.8 × 10^−3^
Stage × risk interaction (n = 504, events = 182)	-	-	0.185
Binary high-vs-low + stage group (n = 504, events = 182)	+0.65	1.91 (1.40–2.61)	4.4 × 10^−5^

Stage-stratified Cox analyses showed HRs of 1.39 (*p* = 0.14), 1.68 (*p* = 0.033), 2.63 (*p* = 0.004), and 2.09 (*p* = 0.26) for stages I–IV, respectively; the stage IV estimate was based on only 25 patients. Aggregated HRs were 1.58 (95% CI: 1.15–2.17; *p* = 4.9 × 10^−3^) in early-stage disease and 2.44 (95% CI: 1.40–4.27; *p* = 1.8 × 10^−3^) in late-stage disease. The stage × risk interaction was not significant (*p* = 0.185), although point estimates were larger in later-stage groups.

Random-effects meta-analysis of the continuous-risk score across GSE50081, GSE68465, and GSE30219 yielded a pooled HR of 1.42 (95% CI: 1.15–1.75; *p* = 1.0 × 10^−3^; I^2^ = 24.9%; τ^2^ = 0.009) (Supplementary Material S2.2). Binary high-versus low-risk grouping gave a pooled HR of 1.35 (95% CI: 1.13–1.61; *p* = 8.4 × 10^−4^; I^2^ = 0). LOCO sensitivity preserved the adverse-risk direction, although exclusion of GSE68465 attenuated the continuous-score estimate toward the null (HR 1.26; *p* = 0.054).

In the post-locking TCGA analysis, the stored out-of-fold reference model score remained associated with overall survival after categorical TNM adjustment (HR per standard deviation increase 2.32, 95% CI: 1.39–3.87; *p* = 0.00135; n = 496, 180 events) and improved model fit relative to TNM alone (likelihood-ratio *p* = 0.00110). Optimism-corrected AUCs for TNM, the molecular score, and the combined model were 0.686, 0.722, and 0.715 at 3 years and 0.677, 0.698, and 0.693 at 5 years. Combined-model Brier scores were 0.206 and 0.218, calibration slopes were 1.06 and 0.98, and expected-to-observed ratios were 0.98 and 1.00 at 3 years and 5 years, respectively (Supplementary Figure S3; Supplementary Table S4). Exploratory 3- and 5-year clinicogenomic nomograms are shown in Supplementary Figure S4.

Decision-curve analysis showed maximum incremental net benefits of 0.039 at 3 years and 0.015 at 5 years, with positive incremental benefit confined to threshold probabilities of 0.197–0.600 and 0.394–0.541, respectively (Figure 4). Continuous NRI excluded the null at 3 years but not 5 years, whereas IDI excluded the null at both horizons (Supplementary Figure S5; Supplementary Table S5). Low-risk patients had longer restricted mean survival by 200.6 days at 3 years and 406.3 days at 5 years. The molecular score remained associated with subsequent survival at the 12-month landmark (HR per standard deviation 2.22, 95% CI: 1.19–4.14; *p* = 0.0126), whereas the 24- and 36-month estimates were imprecise. Stage-stratified analyses retained the overall adverse-risk direction but had wider confidence intervals in smaller strata (Supplementary Figures S6 and S7; Supplementary Table S6).

**FIGURE 4:**
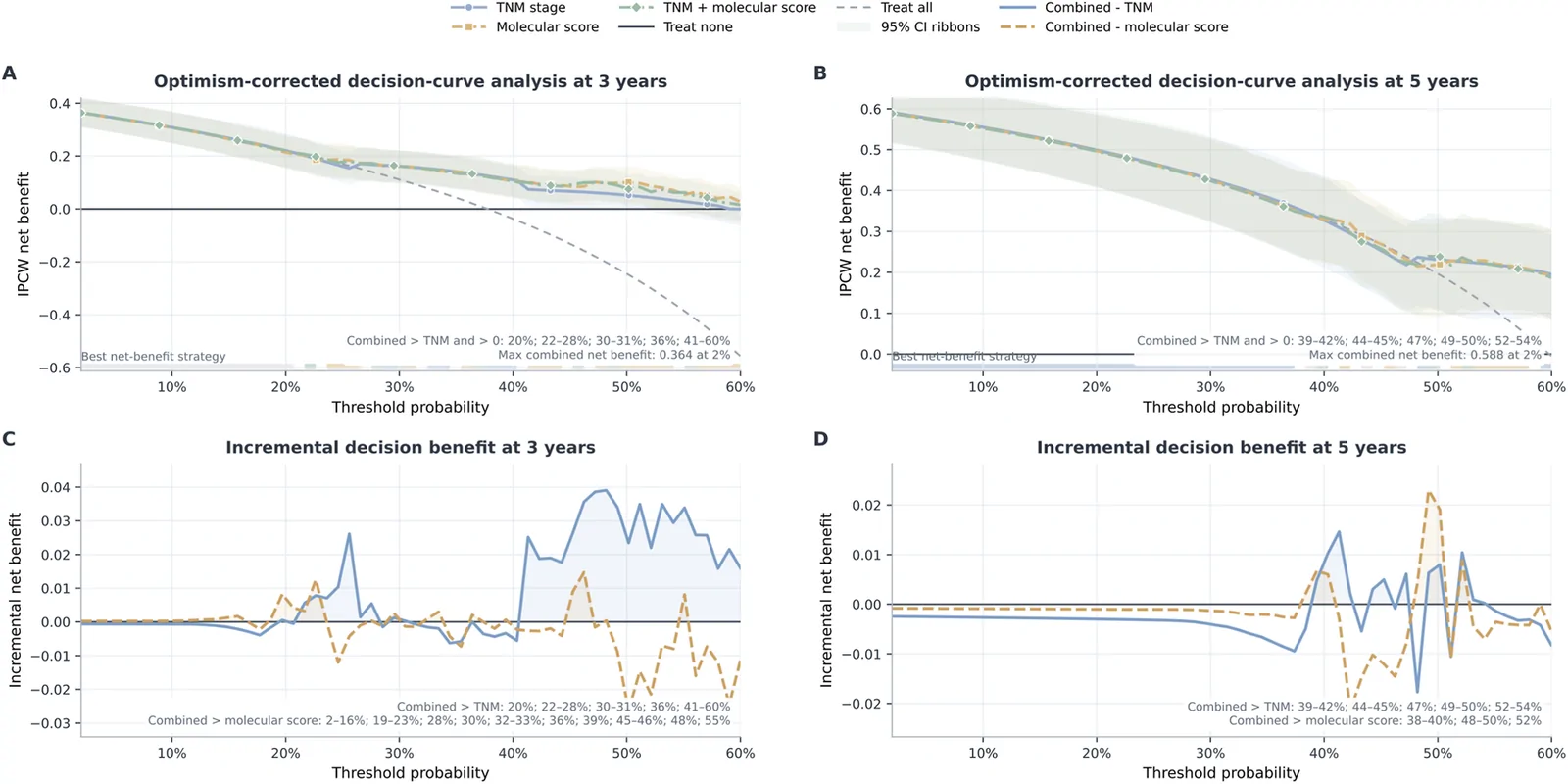
Bootstrap optimism-corrected decision-curve analysis at 3 and 5 years. **(A)** Optimism-corrected decision-curve analysis at 3 years showing IPCW net benefit for TNM stage, the molecular score, and the combined TNM-plus-molecular-score model across threshold probabilities from 0.02 to 0.60; treat-all and treat-none strategies are shown as reference policies. **(B)** Corresponding optimism-corrected decision-curve analysis at 5 years. Shaded bands in **(A,B)** represent bootstrap optimism-corrected 95% confidence intervals. **(C)** Incremental net benefit at 3 years for the combined model relative to TNM stage alone and the molecular score alone. **(D)** Corresponding incremental net benefit at 5 years. The combined model provided positive incremental net benefit over restricted threshold ranges, with a maximum incremental net benefit of 0.039 at 3 years and 0.015 at 5 years; the benefit was more limited at the 5-year horizon.

### 3.4 Functional, network, and microenvironmental characterization of the prioritized gene set

Over-representation analysis (ORA) of the 50-gene set revealed a dominant immune/inflammatory signal (Figure 5). The top Gene Ontology (GO) Biological Process terms included leukocyte adhesion, lymphocyte differentiation, and B-cell activation. KEGG enrichment highlighted necroptosis and cytokine-receptor interaction, whereas Reactome enrichment captured interleukin and IFN-γ signaling cascades. The leading MSigDB Hallmark terms were allograft rejection, interferon-γ response, TNF/NF-κB signaling, and inflammatory response (p_adj_ ≤ 1 × 10^−5^). Direction-stratified enrichment placed protective genes on the adhesion/differentiation axis and risk genes on pyroptotic hubs (IL1B, CASP1, CXCL10, IRF1). STRING database yielded one connected component with consensus hubs (IL1B, CXCL10, IRF1, CASP1, PTPRC, CCR7, CD40LG) and two Louvain communities; an adaptive-immunity block (CCR7/CD28/IL7R/BTK/FYN) and an innate IFN-γ block (AIM2/CASP1/IRF1/RIPK2/TLR2); hub-targeted node removal disrupted connectivity faster than random. Across all six cohorts, low-risk tumors showed a higher ImmuneScore (r = 0.900), CD8^+^ (r = 0.605) and CD4^+^ T cells, B cells, DCs, and M1 macrophages, with reciprocally lower purity (r = −0.845), consistent with an immune-inflamed phenotype linkable to ferroptosis-T-cell crosstalk (Lang et al., 2019; Wang et al., 2019) (Figures 5, 6).

**FIGURE 5:**
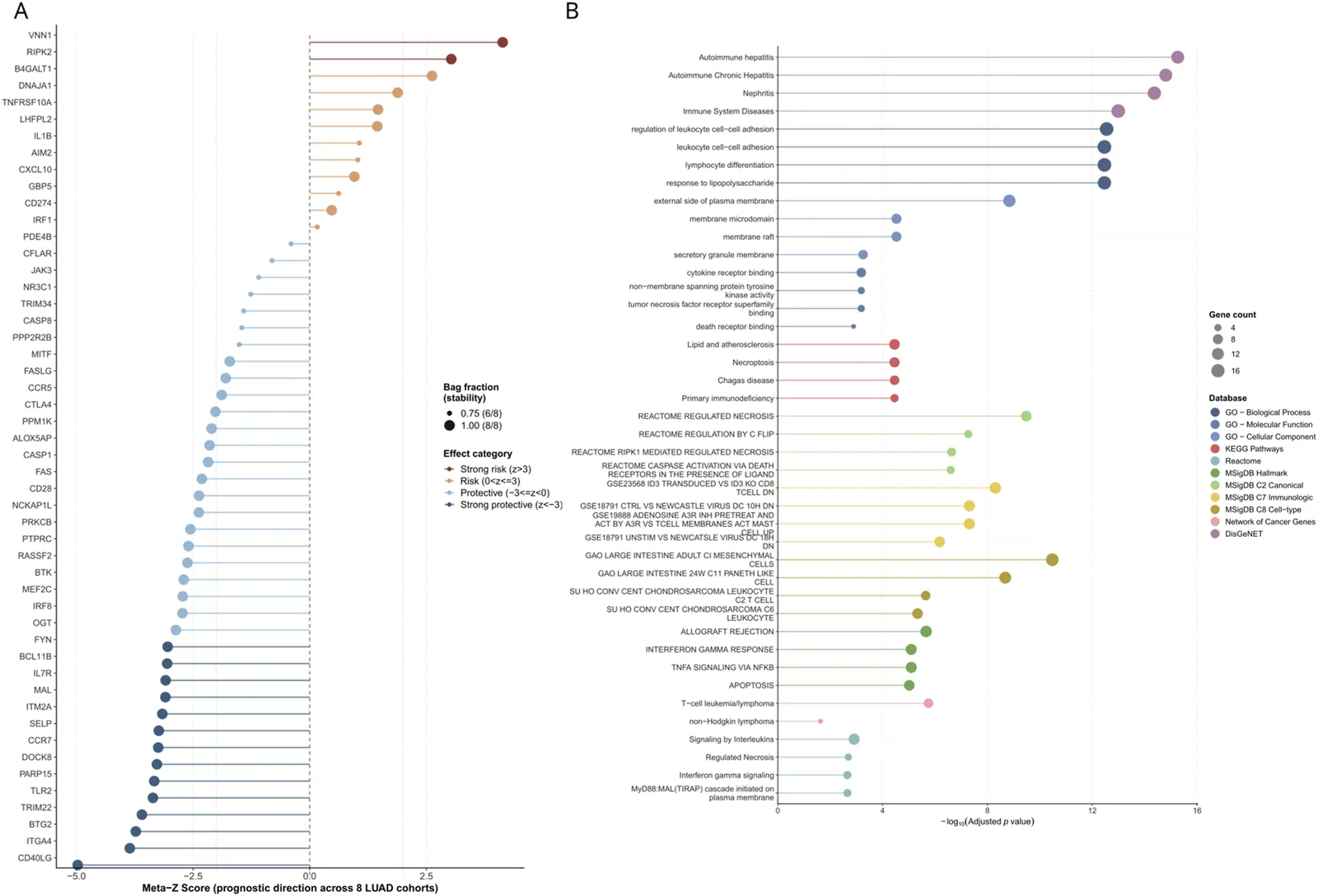
**(A)** Prognostic direction and pathway enrichment of the extended ferroptosis-linked signature. **(B)** Enriched terms converged on leukocyte adhesion, lymphocyte differentiation, interferon signaling, TNF/NF-κB signaling, regulated necrosis, and pyroptosis.

**FIGURE 6:**
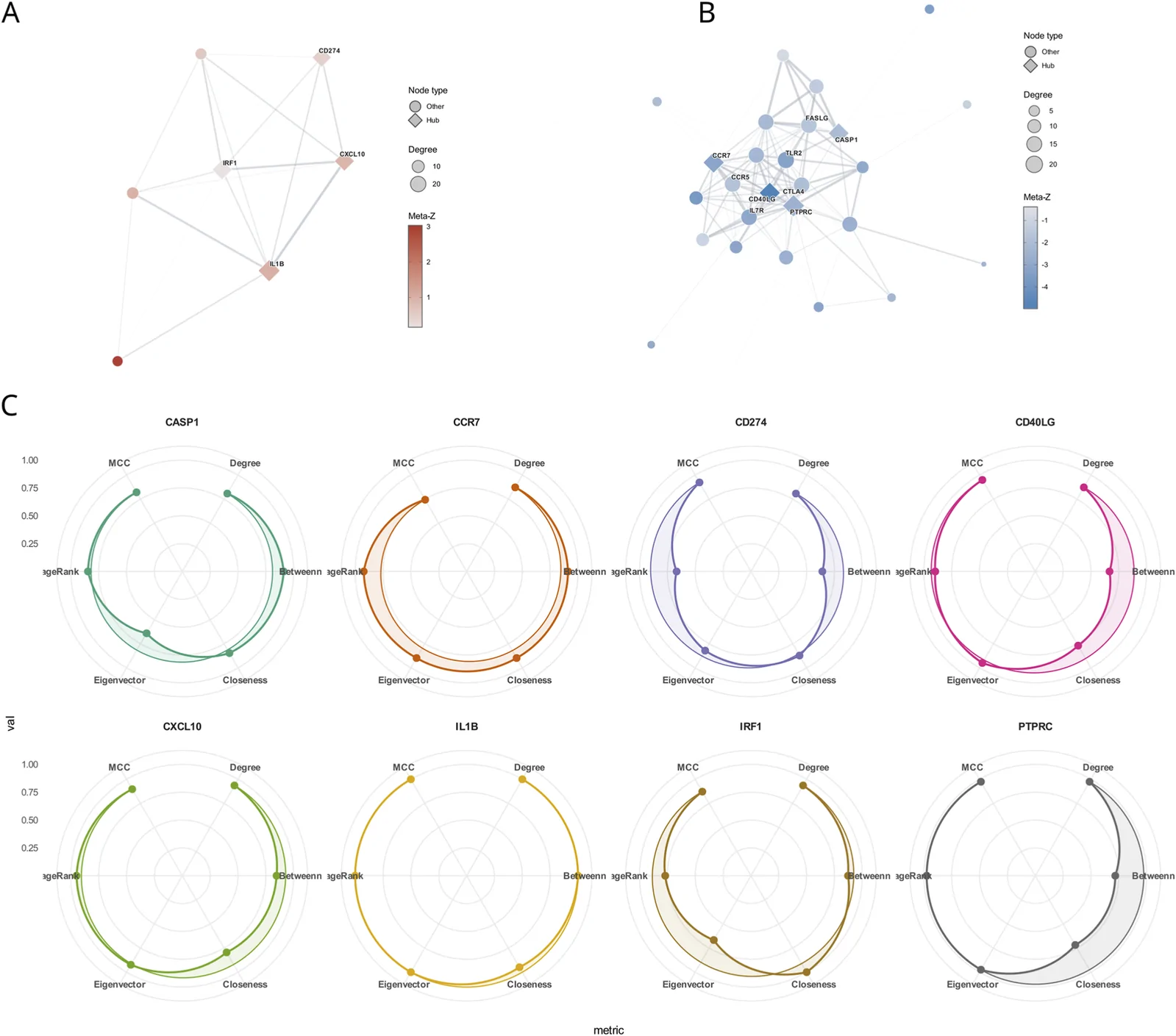
**(A,B)** STRING protein–protein interaction subnetworks and hub-gene centrality profiles. Risk-associated and protective/immune-associated subnetworks: node color = cross-cohort meta-Z direction; node size = degree; diamond nodes = consensus hubs; edge thickness = interaction confidence. **(C)** Lower radar plots: normalized centrality metrics (degree, betweenness, closeness, eigenvector, PageRank, and maximal clique centrality). The network highlights an innate-inflammatory risk module (IL1B, CXCL10, IRF1, CASP1), a protective immune-receptor module (CCR7, CD40LG, PTPRC, and related T-cell signaling genes).

### 3.5 Supportive interpretability and translational analyses

Cross-cohort surrogate interpretation showed moderate but significant feature-ranking concordance (Kendall’s W = 0.541, *p* = 0.0038) and a distributed multigene architecture (Supplementary Figure S8; Supplementary Table S7). Latent immunotherapy-propensity analyses identified interferon-, complement-, and inflammatory-enriched states, but no cohort contained observed immune-checkpoint-inhibitor response labels. Druggability mapping across DGIdb (Freshour et al., 2021), ChEMBL (Zdrazil et al., 2024), and Open Targets (Ochoa et al., 2023), together with structural triage using UniProt (Consortium, 2015), PDB, and AlphaFold (Varadi et al., 2022), nominated follow-up hypotheses including RIPK2, OGT, FYN, CCR7, and CD40LG (Supplementary Material S2.3, Supplementary Figure S9; Supplementary Table S8).

### 3.6 Signature robustness and comparison with published signatures

Twenty-four of the 25 locked genes were selected in all three LOCO folds, and the minimum selection frequency was 0.67. With a 25-gene cap, perturbing the stored LOCO-frequency rule retained 23 genes; increasing the cap to 30 retained 24. Independent re-selection under alternative directional-consistency and cohort-contribution rules recovered 24%–56% of the panel, indicating high fold-level persistence but only partial invariance to broader selection changes. Among the ten locked genes measurable in GSE136961, exclusion of this small cohort preserved all effect directions and produced a Spearman correlation of 0.964 for meta-Z estimates. Algorithm-family profiles across 150 configurations supported the development-selected extra survival trees reference model while showing lower discrimination for several alternative families (Supplementary Material S2.4, Supplementary Figures S10 and S11; Supplementary Tables S9 and S10).

## 4 Discussion

This study presents a biologically constrained and leakage-controlled framework for identifying a transportable ferroptosis-linked prognostic program across heterogeneous lung adenocarcinoma (LUAD) cohorts. Independent WGCNA/FerrDb candidate restriction, fold-local feature selection, hyperparameter tuning, LOCO stability locking, and reserved external evaluation were used to limit optimistic performance estimation. The resulting model showed moderate external performance, including cross-histology assessment in GSE30219, while fixed-horizon analyses indicated limited incremental clinical value beyond TNM stage. These findings support further prospective assay-level evaluation before clinical application.

Previous LUAD ferroptosis signatures have linked ferroptosis-related genes with survival, immune infiltration, mutational features, and drug-response surrogates (Ma et al., 2021; Ren et al., 2021; Zhang Z. et al., 2021; Zhu et al., 2021). The framework presented here extends this work by separating biological candidate definition, fold-local multi-cohort development, panel locking, and external evaluation. Published signatures have generally relied on single-cohort discovery followed by validation in one or a limited number of datasets. Because endpoints, time horizons, cohort composition, and validation designs vary, reported AUCs and C-indices are presented descriptively (Supplementary Figure S12; Supplementary Table S11).

The selected genes were not dominated by canonical ferroptosis-execution genes such as GPX4, SLC7A11, and FSP1/AIFM2. Instead, they represent a broader network involving lipid peroxidation, redox buffering, iron handling, and inflammatory signaling (Chen et al., 2021; Ito et al., 2024; Lei et al., 2022), together with glutathione/selenium metabolism, mitochondrial state, and cellular stress responses (Dixon et al., 2012; Doll et al., 2019; Jiang et al., 2021; Yang et al., 2014). CCR7 and IL7R are involved in immune trafficking and lymphocyte biology (Hong et al., 2022; Barata et al., 2019), whereas PTPRC, FYN, CD28, and CTLA4 contribute to antigen-receptor and co-stimulatory signaling (Rheinländer et al., 2018; Esensten et al., 2016). CXCL10, CD40LG and RIPK2 reflect interferon and inflammatory activation (Tokunaga et al., 2018; Elgueta et al., 2009; Caruso et al., 2014), FAS and CASP1 connect the panel to death-receptor and inflammasome pathways (Strasser et al., 2009; Shi et al., 2015), and ALOX5AP links it to ferroptosis-adjacent lipid-peroxidation stress (Mashima and Okuyama, 2015). The signature therefore appears to capture a ferroptosis-associated immune-inflammatory state rather than ferroptotic flux itself.

The immune-context results are consistent with established ferroptosis–immune interactions. Activated CD8^+^ T cells can promote tumor-cell ferroptosis through interferon-γ-mediated repression of SLC3A2 and SLC7A11 (Wang et al., 2019), while radiotherapy and immunotherapy may converge on SLC7A11 suppression (Lang et al., 2019). The observed interferon-enriched pattern also resembles a tumor-inflammation expression signature associated with pembrolizumab response (Ayers et al., 2017), although treatment-response data were unavailable in the analyzed cohorts. Ferroptosis may also impair anti-tumor immunity because tumor-infiltrating CD8^+^ T cells can undergo lipid-peroxidation-driven ferroptosis (Drijvers et al., 2021). Thus, the inferred state may reflect either productive anti-tumor immunity or maladaptive inflammatory stress (Friedmann Angeli et al., 2019). VNN1, B4GALT1, and DNAJA1 further connect the signature with oxidative stress, adhesion, and mutant-p53/NF-κB biology (Kang et al., 2016; Cheng et al., 2023; Wu et al., 2023).

Multi-method immune deconvolution reduced dependence on a single computational method (Zeng et al., 2024; 2021), and network and surrogate-SHAP analyses indicated that prognostic information was distributed across the program (Mantovani et al., 2018; Gentles et al., 2015). Structural prioritization identified candidates suitable for experimental follow-up (Varadi et al., 2022). However, computational attribution and target ranking alone do not establish causal or therapeutic effects.

The transcriptomic and clinical data were obtained from public repositories and were not complemented by an independent in-house tissue cohort, targeted RNA or protein assays, spatial validation, or causal cellular and animal experiments. Platform heterogeneity and incomplete gene coverage constrained the small-cohort exclusion analysis, while the mixed-histology composition of GSE30219 limits LUAD-specific inference. Although fold-level persistence was high, independent re-selection recovered only part of the panel, indicating sensitivity to analytical and cohort variation. Clinical translation will require population-specific recalibration, predefined assay cut-points, and prospective validation. Further studies should prioritize assay standardization, independent clinically annotated cohorts, spatial or single-cell localization, and perturbation experiments that directly quantify ferroptotic flux and immune-cell causality.

## Data Availability

All scripts, random seeds, session-information logs, output tables, figures, and checkpoint files are deposited at https://github.com/salihoglu/ML---Lung-Cancer. Public datasets used in this study are TCGA-LUAD (GDC), GSE31210, GSE72094, GSE136961, GSE50081, GSE68465, GSE30219, and GSE81089 (WGCNA discovery). FerrDb V3 was used as the curated ferroptosis catalogue; STRING v12.0 for the PPI network; MSigDB Hallmark, C2 CP, C7 immunologic, C8 cell-type, and CGP for gene-set/connectivity analyses; ChEMBL, DGIdb, and Open Targets for druggability mapping; UniProt, PDB, and AlphaFold for structural prioritisation.
