# Supplementary material for: Multi-cohort machine learning identifies a ferroptosis-linked prognostic signature in lung adenocarcinoma
Source: Front Bioinform. 2026 Sep 17;6:1921468. doi: 10.3389/fbinf.2026.1921468 (PMC13628503; doi:10.3389/fbinf.2026.1921468)
Supplement: Supplementary file 1 [file DataSheet1.pdf]

SUPPLEMENTARY MATERIAL

Multi-cohort machine learning identifies a ferroptosis-linked prognostic signature in lung adenocarcinoma

Rana Salihoglu[1,2]

[1]Department of Computer Engineering, Recep Tayyip Erdogan University, Rize, Türkiye

[2]Chair of Bioinformatics, Julius-Maximilians-Universität Würzburg, Würzburg, Germany

Correspondence: ranasalihoglu@gmail.com

Supplementary Methods

S1.1 Fixed-configuration external sensitivity

External sensitivity analyses used the complete stored patient-level predictions for GSE50081, GSE68465 and GSE30219. GSE30219 was retained as the complete mixed-histology cohort (n=289; 196 events), and no post hoc histology-defined subset was generated. A fixed-configuration sensitivity analysis applied the development-selected Extra Survival Trees configuration to all three cohorts. Harrell's concordance, 5-year Uno's concordance, 3- and 5-year time-dependent AUC and continuous-score Cox associations were calculated from the same stored risk predictions. Random-effects meta-analysis, leave-one-external-cohort-out analysis, Kaplan-Meier stratification, RMST and landmark analyses used unchanged cohort definitions. These analyses were distinct from the descriptive per-cohort maxima among the prespecified candidate configurations.

S1.2 Fixed-horizon clinical utility, calibration, decision curves and nomograms

The stored out-of-fold molecular score was linked to TCGA-LUAD patients using the original sample ordering and exact survival matching. Relative-hazard inference compared categorical AJCC/TNM stage, the standardised molecular score and their combination in one stage-complete cohort. Cox models used Efron partial likelihood, likelihood-ratio comparisons and Schoenfeld-residual diagnostics with multiplicity correction. Absolute-risk outputs were generated from a coherent fixed-horizon layer at 3 and 5 years. Deaths by the horizon were cases, patients surviving beyond the horizon were controls, and observations censored before the horizon were excluded from the corresponding binary outcome. IPCW-weighted ridge logistic models were fitted for TNM stage, the molecular score and their combination; regularisation was selected by cross-validation, and non-negative coefficients preserved ordered risk direction. Time-dependent AUC, IPCW Brier score, calibration intercept and slope, expected-to-observed ratio, continuous NRI and IDI were obtained from the same predictions. DCA compared TNM, score and combined models with treat-all and treat-none policies at thresholds from 0.02 to 0.60. Horizon-specific nomograms converted combined-model coefficients to total points and overall-survival probability. Calibration, DCA and incremental metrics were internally validated by 1,000-resample bootstrap optimism correction. RMST at 3 and 5 years and conditional landmark analyses at 12, 24 and 36 months provided proportional-hazards-robust summaries.

S1.3 Cross-cohort interpretability and translational prioritisation

Cross-cohort interpretability tested whether machine-learning-prioritised ferroptosis-related genes reproducibly contributed to stored risk-score architecture. These analyses were not used for feature selection, model fitting, threshold optimisation or performance estimation. HGNC-harmonised genes were z-standardised, and an ExtraTrees regressor approximated the stored risk score (Geurts et al., 2006). Fidelity was assessed by cross-validated R^2 and Spearman correlation. SHAP values and permutation importance were integrated into a consensus ranking, with Kendall's W used to quantify cross-cohort concordance (Li, 2022). Calibration was assessed separately using Cox-recalibrated probabilities, Kaplan-Meier bins, Brier scores and calibration slopes (Graf et al., 1999). Immunotherapy-related pathway states were defined from FerrDb V3 annotations (Zhou et al., 2026) and MSigDB Hallmark, C7 and C8 collections (Subramanian et al., 2005), but were interpreted as latent response propensity because observed immune-checkpoint-inhibitor labels were unavailable. Signature-reversal connectivity and druggability used DGIdb (Freshour et al., 2021), ChEMBL (Zdrazil et al., 2024) and Open Targets (Ochoa et al., 2023). Structural prioritisation combined UniProt reviewed status (Consortium, 2015), PDB experimental structures and AlphaFold availability (Varadi et al., 2022). These layers were hypothesis-generating and did not establish causality or therapeutic efficacy.

S1.4 Signature robustness and comparison with published signatures

Robustness analyses used the stored run configuration, fold-level selection frequencies, algorithm leaderboard, out-of-fold predictions and external patient-level risk files without re-running or modifying the original survival-learning analysis. Stored-rule sensitivity varied the minimum LOCO selection frequency (0.33, 0.50, 0.67 and 1.00) and signature-size cap (20, 25 and 30 genes). Independent re-selection varied directional-consistency requirements and the minimum number of contributing development cohorts, with and without GSE136961. Development-cohort jackknife analyses compared gene-level meta-effects after omission of each cohort. Genes absent from a platform were recorded and were not imputed. Algorithm-family robustness summarised all 150 stored configurations from nine model families. Published LUAD ferroptosis-related signatures were compared descriptively by signature size, discovery strategy, model class, development and validation cohorts, leakage control, experimental validation and reported discrimination. Differences in endpoints, horizons, datasets and metrics precluded direct ranking.

Supplementary Results

S2.1 External fixed-configuration and full-cohort sensitivity

The development-selected Extra Survival Trees configuration produced Harrell's C-indices of 0.605, 0.675 and 0.682 and 5-year Uno's C-indices of 0.605, 0.678 and 0.696 in GSE50081, GSE68465 and complete GSE30219, respectively (Supplementary Figure S1; Supplementary Table S3). Continuous-score hazard ratios per standard-deviation increase were 1.29, 1.70 and 1.72, yielding a random-effects pooled HR of 1.59 (95% CI 1.36-1.85; $p=5.07\times10^{-9}$; $I^2=65.4\%$). Leave-one-external-cohort-out estimates remained significant, and complete-cohort Kaplan-Meier, RMST and landmark analyses retained the same adverse-risk direction (Supplementary Figure S2).

S2.2 Clinical association, complementary external meta-analysis and fixed-horizon utility

The TCGA-refitted weighted score comprised 26 genes selected post hoc from the full-training 50-gene prognostic candidate panel. This linear score was analytically distinct from the locked 25-gene panel, the development-selected Extra Survival Trees reference predictor and its stored out-of-fold molecular score.

In TCGA-LUAD, the continuous TCGA-refitted weighted score was associated with overall survival in the risk-only Cox model (β=0.597; HR 1.82, 95% CI 1.39–2.37; $p=1.1\times10^{-5}$). In the clinicopathologic-only model, late-stage disease (III–IV vs I–II) was the dominant clinical predictor (HR 2.47, 95% CI 1.74–3.50; $p=3.7\times10^{-7}$), whereas age, sex and M-stage were not statistically significant. After adjustment for these variables, the weighted score remained associated with survival (β=0.612; HR 1.84, 95% CI 1.38–2.47; $p=4.3\times10^{-5}$), and late-stage disease remained independently prognostic (HR 2.34, 95% CI 1.65–3.32; $p=2.0\times10^{-6}$). The limited change in the score coefficient between the unadjusted and adjusted models indicated little attenuation after clinical adjustment. Addition of the score improved model fit relative to the clinicopathologic-only model (likelihood-ratio $p=3.9\times10^{-5}$). Median-dichotomised high-risk status was similarly associated with poorer survival after stage adjustment (HR 1.91, 95% CI 1.40–2.61; $p=4.4\times10^{-5}$).

Time-dependent AUCs for the combined weighted-score plus clinicopathologic model were 0.708, 0.668 and 0.654 at 1, 3 and 5 years, respectively, compared with 0.629, 0.617 and 0.632 for the clinicopathologic-only model. These estimates represented the complementary TCGA-refitted association model and should not be interpreted as the performance of the locked external-validation predictor.

Stage-stratified analyses yielded HRs of 1.39 (95% CI 0.90–2.14; p=0.14) in stage I, 1.68 (95% CI 1.04–2.70; p=0.033) in stage II, 2.63 (95% CI 1.37–5.05; p=0.004) in stage III and 2.09 (95% CI 0.58–7.51; p=0.26) in stage IV. The stage IV estimate was based on 25 patients and 16 events and consequently had a wide confidence interval. Aggregated analyses produced HRs of 1.58 (95% CI 1.15–2.17; $p=4.9\times10^{-3}$) in stages I–II and 2.44 (95% CI 1.40–4.27; $p=1.8\times10^{-3}$) in stages III–IV. However, the stage × score interaction was not significant (p=0.185), providing no statistical evidence that the score effect differed by stage.

For the complementary external association analysis, the continuous TCGA-refitted weighted score was re-fitted separately by Cox regression in GSE50081, GSE68465 and the complete mixed-histology GSE30219 cohort. Random-effects meta-analysis yielded a pooled HR of 1.42 per score-unit increase (95% CI 1.15–1.75; $p=1.0\times10^{-3}$; I^2=24.9%; τ^2=0.009). Median-defined high- versus low-risk grouping produced a pooled HR of 1.35 (95% CI 1.13–1.61; $p=8.4\times10^{-4}$; I^2=0%). Leave-one-cohort-out sensitivity analyses preserved the adverse-risk direction, although omission of GSE68465 attenuated the continuous-score estimate toward the null (HR 1.26; p=0.054). These cohort-specific Cox refits were complementary association analyses and were distinct from the unchanged fixed-reference ETX evaluation reported in Supplementary Results S2.1.

The post-locking clinical-utility analysis used the stored out-of-fold score generated by the development-selected Extra Survival Trees reference configuration. The score was linked to stage-complete TCGA-LUAD cases using the original sample ordering and exact survival matching. The resulting analysis included 496 patients and 180 deaths.

After adjustment for categorical TNM stage, each standard-deviation increase in the stored molecular score was associated with poorer overall survival (HR 2.32, 95% CI 1.39–3.87; p=0.00135). Adding the score to TNM stage improved Cox-model fit relative to TNM alone (likelihood-ratio p=0.00110). Because Schoenfeld-residual diagnostics did not uniformly support proportional stage effects, absolute-risk performance was evaluated separately at 3 and 5 years using IPCW-weighted fixed-horizon models.

At 3 years, the optimism-corrected AUC was 0.686 (95% CI 0.622–0.749) for TNM stage, 0.722 (95% CI 0.661–0.788) for the molecular score and 0.715 (95% CI 0.652–0.776) for the combined model. The corresponding IPCW Brier scores were 0.212 (95% CI 0.195–0.237), 0.204 (95% CI 0.184–0.229) and 0.206 (95% CI 0.186–0.232). Calibration slopes were 1.125 (95% CI 0.727–1.737), 1.023 (95% CI 0.742–1.458) and 1.060 (95% CI 0.759–1.488), whereas

expected-to-observed ratios were 0.985 (95% CI 0.845–1.121), 0.975 (95% CI 0.834–1.106) and 0.977 (95% CI 0.850–1.109), respectively. All 1,000 bootstrap resamples were successful for the TNM and score-only models, compared with 779 successful combined-model refits.

At 5 years, optimism-corrected AUCs were 0.677 (95% CI 0.606–0.752) for TNM stage, 0.698 (95% CI 0.619–0.782) for the molecular score and 0.693 (95% CI 0.615–0.778) for the combined model. The corresponding Brier scores were 0.220 (95% CI 0.202–0.246), 0.215 (95% CI 0.196–0.242) and 0.218 (95% CI 0.197–0.246). Calibration slopes were 1.178 (95% CI 0.581–2.257), 1.163 (95% CI 0.710–1.956) and 0.979 (95% CI 0.616–1.642), and expected-to-observed ratios were 0.995 (95% CI 0.884–1.113), 0.999 (95% CI 0.888–1.114) and 1.000 (95% CI 0.878–1.113), respectively. The TNM and score-only models completed all 1,000 bootstrap resamples, whereas 608 combined-model refits were successful. The reduced number of successful combined-model refits at 5 years indicates greater resampling instability and supports cautious interpretation of the internally validated 5-year estimates (Supplementary Figure S3; Supplementary Table S4). The corresponding exploratory clinicogenomic nomograms are shown in Supplementary Figure S4.

Although the combined model modestly outperformed TNM alone, the molecular-score-only model showed slightly higher AUC and lower Brier score than the combined model at both horizons. Relative to TNM alone, adding the molecular score increased AUC by 0.029 at 3 years and 0.016 at 5 years and improved the Brier score by 0.0066 and 0.0019, respectively (Supplementary Table S5).

The maximum incremental net benefit of the combined model relative to TNM was 0.0391 at 3 years and 0.0146 at 5 years. Positive incremental benefit occurred only at restricted, non-contiguous thresholds within the overall probability spans of 0.197–0.600 and 0.394–0.541, respectively, rather than throughout these entire intervals. Thus, decision-curve analysis supported only limited and horizon-dependent incremental utility (Supplementary Figure S5).

Continuous NRI was 0.499 (95% CI 0.256–0.724) at 3 years and 0.306 (95% CI −0.022–0.606) at 5 years. The 3-year interval excluded zero, whereas the 5-year interval did not. IDI estimates were 0.0325 (95% CI 0.0152–0.0480) and 0.0282 (95% CI 0.0091–0.0455) at 3 and 5 years, respectively, with both intervals excluding zero. These reclassification estimates were derived from the same horizon-specific predictions used for discrimination, calibration and decision-curve analysis (Supplementary Figure S5; Supplementary Table S5). These nomograms represent internally derived risk summaries and have not undergone external recalibration, assay-transfer evaluation or prospective validation.

Low-risk patients had longer restricted mean survival than high-risk patients by 200.6 days over 3 years and 406.3 days over 5 years. In conditional landmark analyses, the molecular score remained associated with subsequent survival among patients alive at 12 months (HR per standard-deviation increase 2.22, 95% CI 1.19–4.14; p=0.0126). The corresponding estimates at 24 months (HR 1.75; p=0.157) and 36 months (HR 0.70; p=0.466) were imprecise and did not provide evidence of a persistent conditional association at these later landmarks. Stage-stratified analyses retained the overall adverse-risk direction but produced wider confidence intervals in smaller subgroups (Supplementary Figures S6 and S7; Supplementary Table S6).

S2.3 Interpretability and translational prioritisation

Feature-ranking concordance across GSE30219, GSE50081 and GSE68465 was moderate but significant (Kendall's W=0.541, p=0.0038), supporting a distributed multigene risk architecture (Supplementary Figure S8). RIPK2 showed the strongest consensus support, followed by MEF2C, MAL, ITM2A, CXCL10, TLR2, OGT, LHFPL2, VNN1 and CD40LG (Supplementary Table S7). Benefit-High samples were enriched for interferon, complement and inflammatory

programmes, but these scores did not represent observed treatment response. Druggability and structural triage nominated PARP15, BTK, OGT, FYN, RIPK2, CCR7 and CD40LG as Tier-A follow-up targets, with PTPRC, ITGA4 and TLR2 assigned Tier B (Supplementary Figure S9; Supplementary Table S8). Candidate compounds and targets remain hypothesis-generating.

S2.4 Signature robustness and literature comparison

Twenty-four of the 25 locked genes were selected in all LOCO folds, and the minimum selection frequency was 0.67. Stored-rule perturbation retained 23 of 25 genes at a 25-gene cap and 24 of 25 at a 30-gene cap (Supplementary Figure S10; Supplementary Table S9). Independent alternative re-selection retained 24-56% of the panel. Among the ten locked genes measurable in GSE136961, exclusion of the small cohort preserved all directions and produced Spearman $\rho=0.964$ for meta-Z estimates (Supplementary Figure S11; Supplementary Table S10). Published signatures generally used single-cohort discovery followed by limited external validation, whereas the present study separated the biological gate, fold-local multi-cohort development, stability locking and reserved external evaluation. Reported performance is shown descriptively and is not a head-to-head comparison (Supplementary Figure S12; Supplementary Table S11).

Supplementary Figures

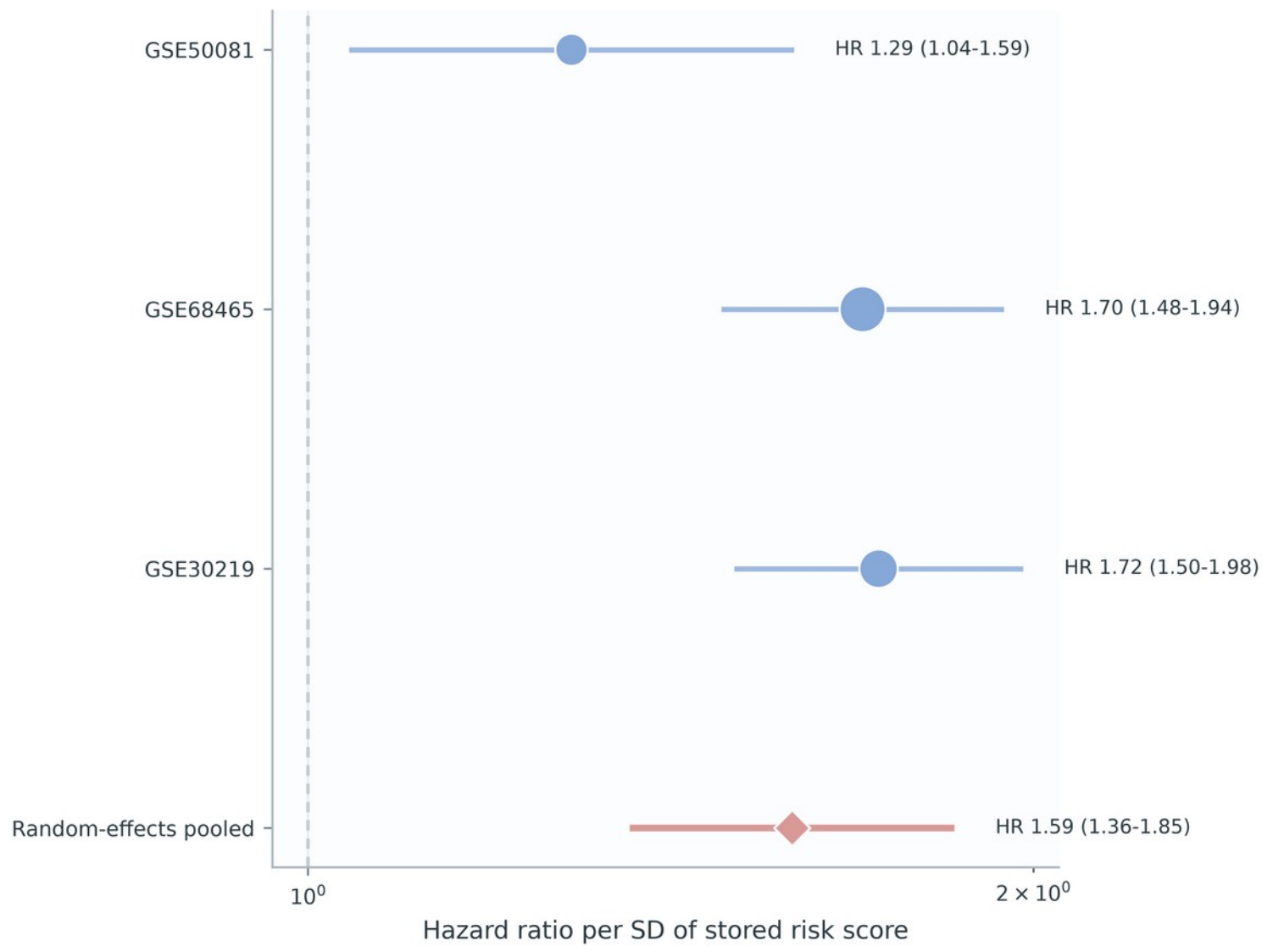

Supplementary Figure S1. Fixed-configuration external evaluation using the complete stored cohorts. Cohort-specific hazard ratios per standard-deviation increase in the development-selected Extra Survival Trees score and the random-effects pooled estimate are shown for GSE50081, GSE68465 and complete GSE30219. GSE30219 was retained as the complete mixed-histology cohort.

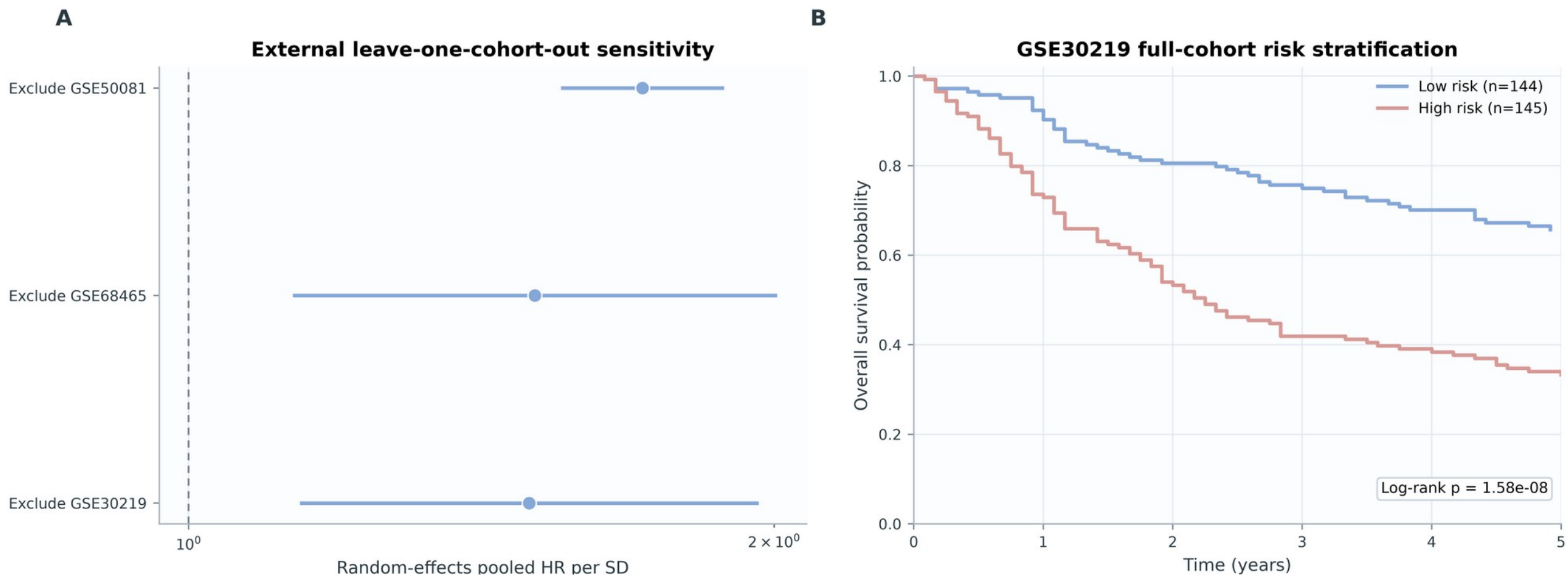

Supplementary Figure S2. Complete-cohort external sensitivity. **(A)** Leave-one-external-cohort-out random-effects estimates. **(B)** Kaplan-Meier stratification in GSE30219. Complementary RMST and landmark analyses used the same cohort definitions; no histology-defined subset was generated.

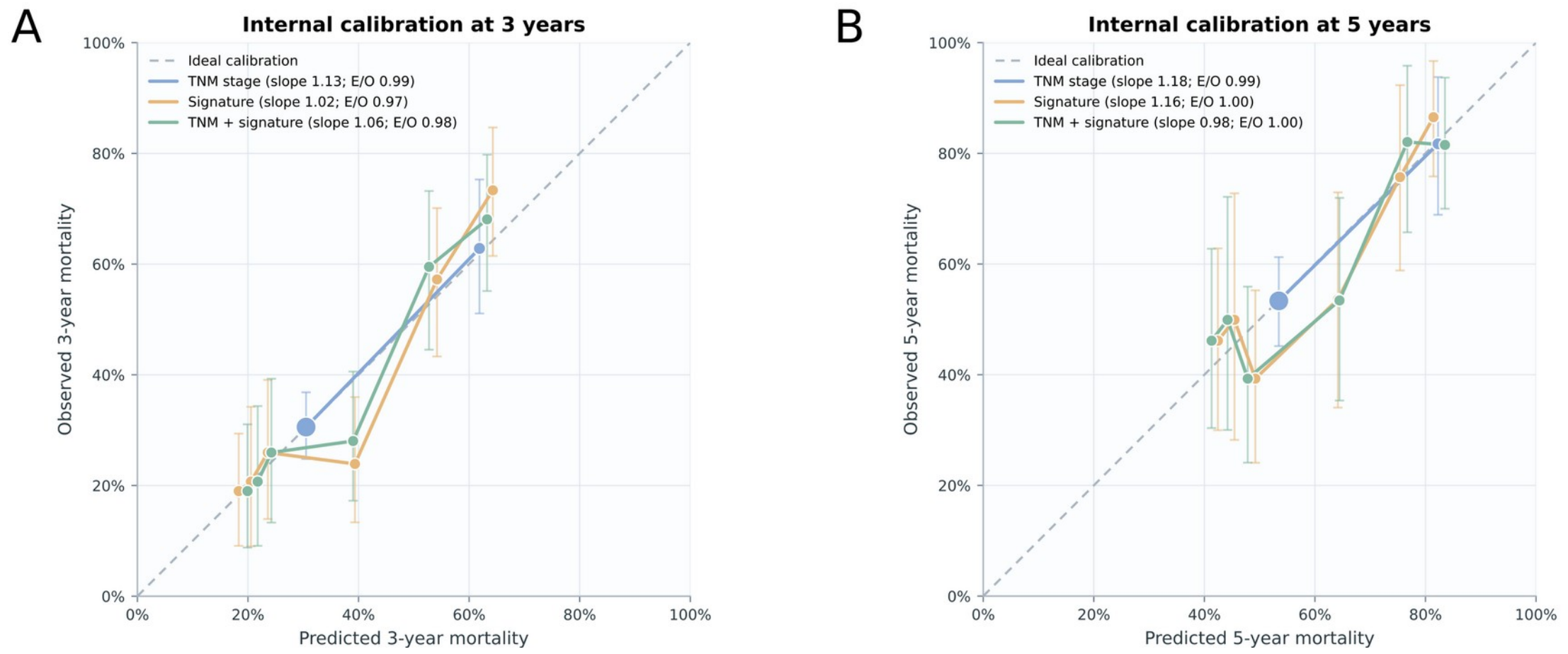

Supplementary Figure S3. Internal calibration at 3 and 5 years. Grouped Kaplan-Meier observed mortality with bootstrap 95% confidence intervals is plotted against predicted mortality for TNM stage, the stored out-of-fold molecular score and their combination. The diagonal denotes ideal calibration; marker size reflects group size.

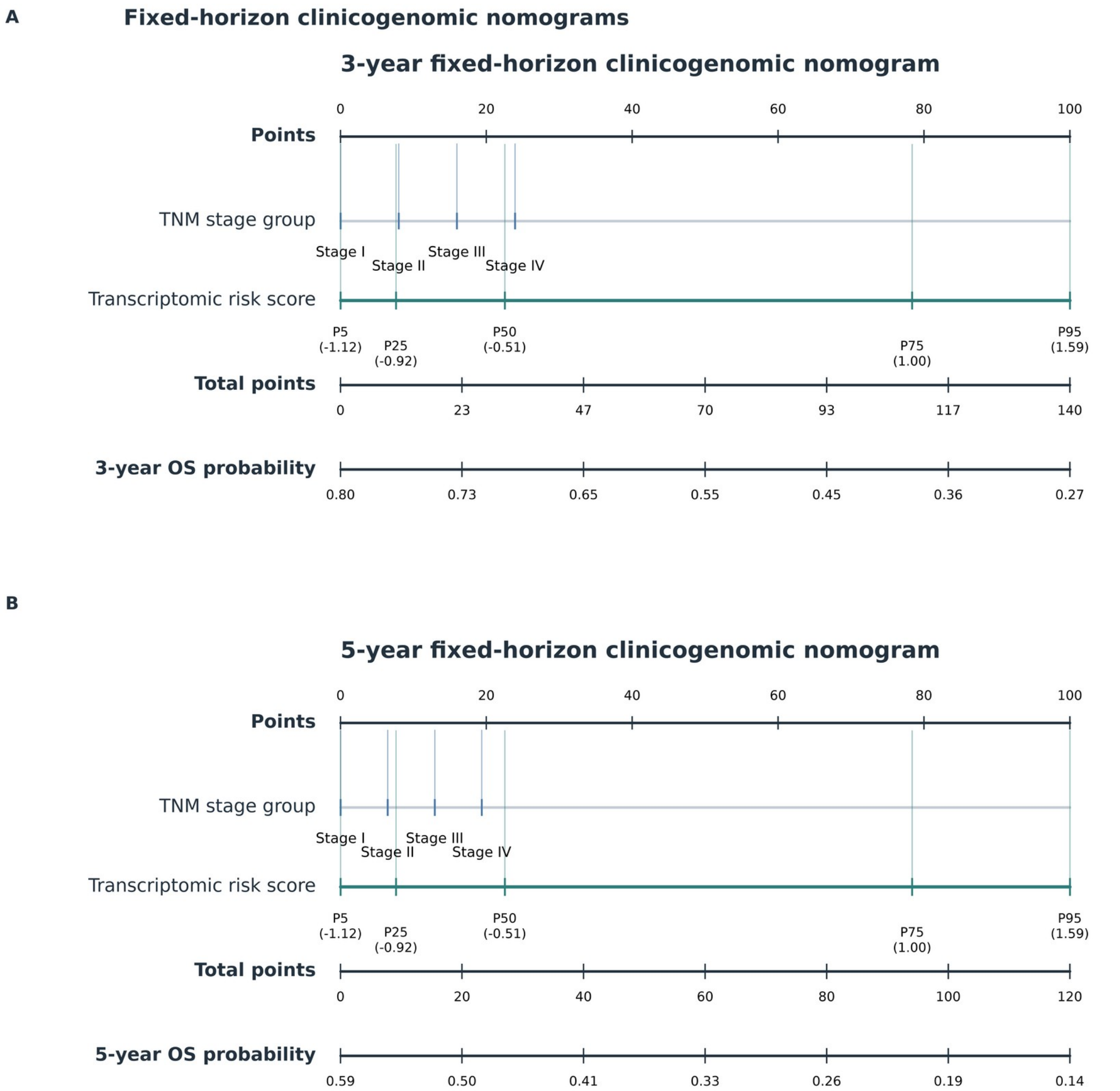

Supplementary Figure S4. Exploratory 3- and 5-year fixed-horizon clinicogenomic nomograms. Ordered TNM stage and the stored molecular score contribute to total points, which map to horizon-specific overall-survival probability. The nomograms require assay transfer, external recalibration and prospective validation before patient-level use.

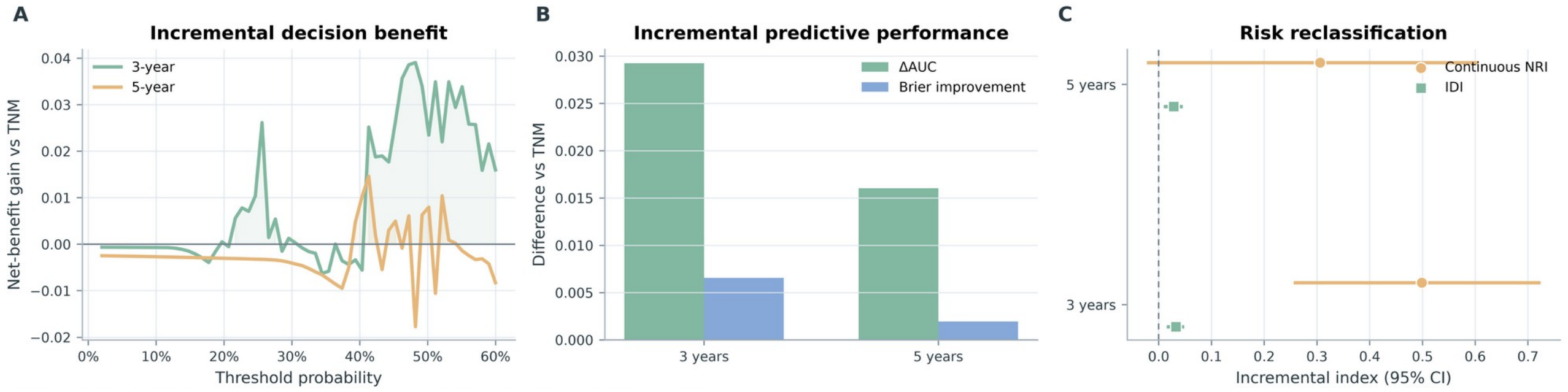

Supplementary Figure S5. Incremental clinical value of adding the molecular score to TNM stage. **(A)** Difference in IPCW net benefit. **(B)** Change in time-dependent AUC and Brier-score improvement. **(C)** Continuous NRI and IDI with bootstrap 95% confidence intervals. Intervals crossing zero are interpreted as inconclusive.

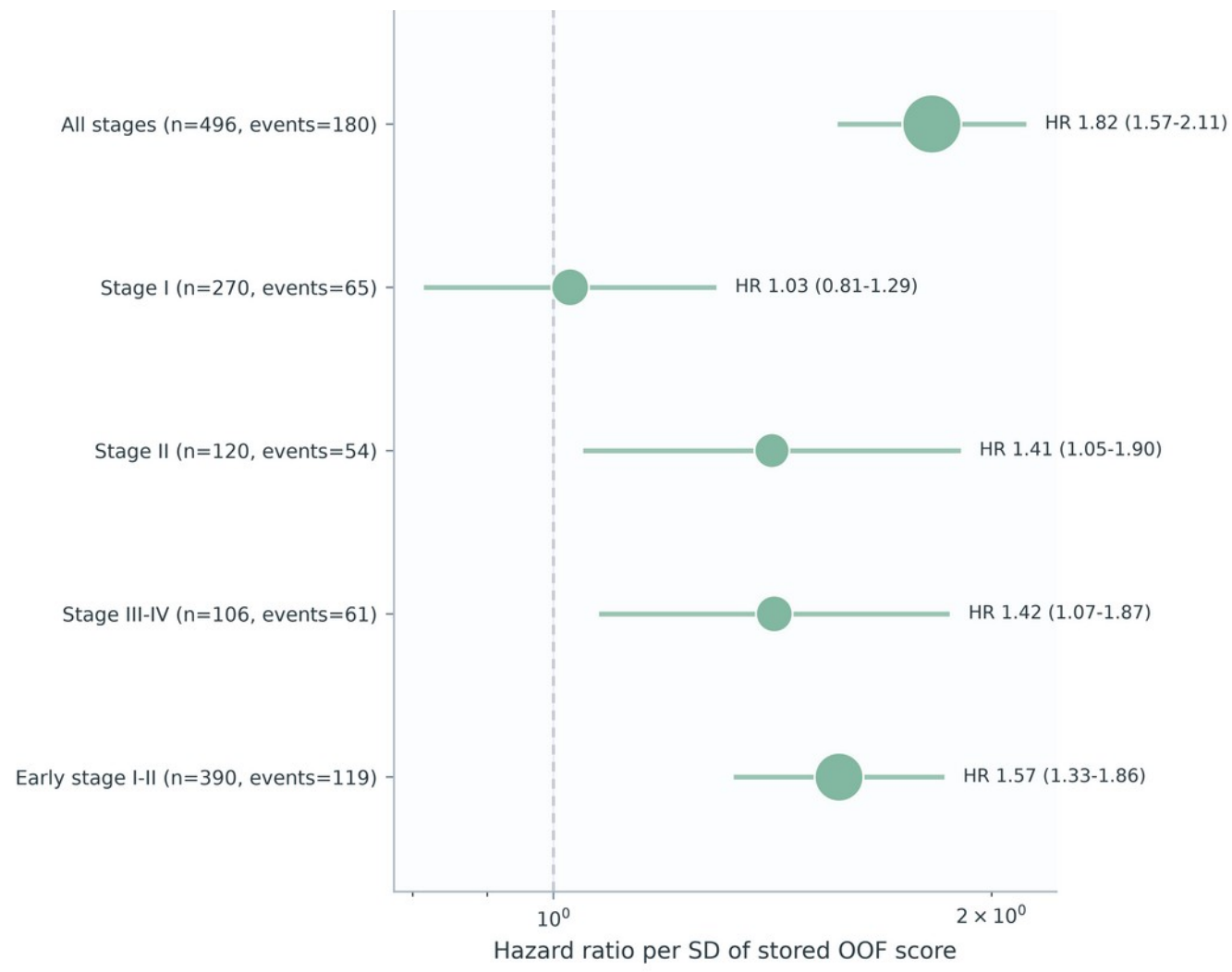

Supplementary Figure S6. Stage-stratified association of the stored molecular score. Hazard ratios per standard-deviation increase are shown overall and by pathological-stage strata. Confidence intervals widen in smaller strata; the analysis is descriptive and complements the primary stage-adjusted model.

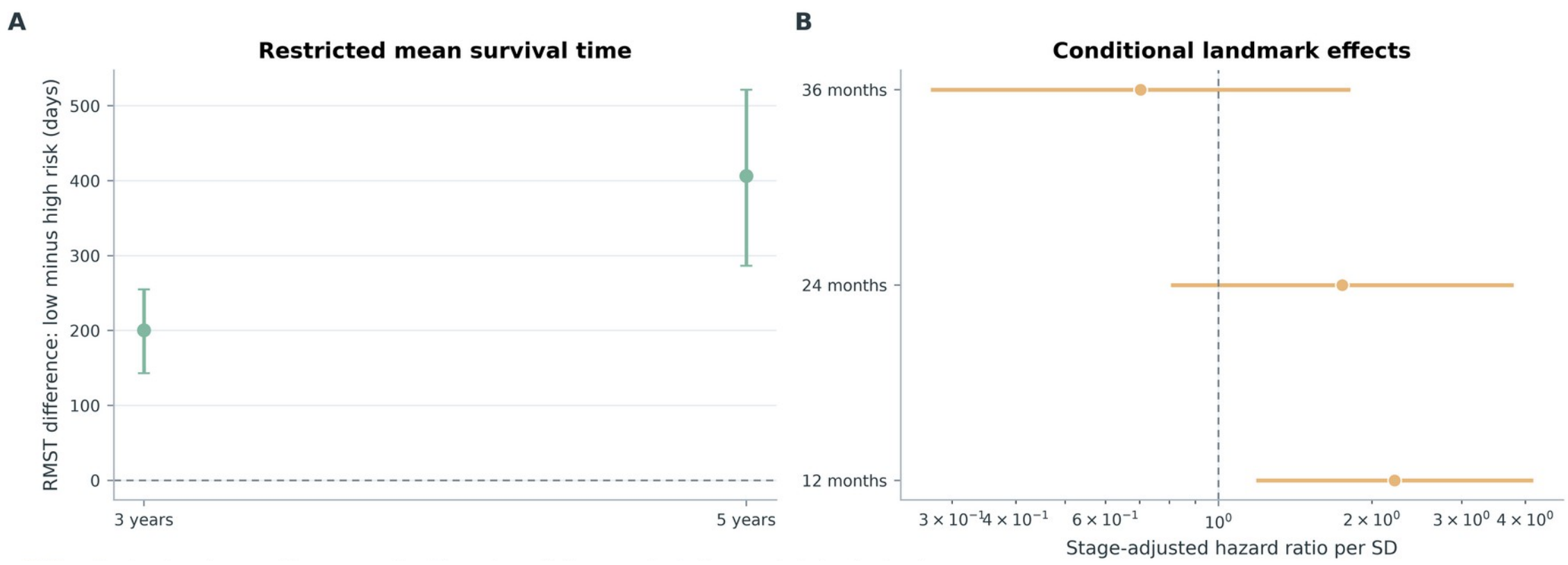

Supplementary Figure S7. Proportional-hazards-robust survival sensitivity. (A) Restricted mean survival-time differences between low- and high-risk groups at 3 and 5 years. (B) Conditional landmark hazard ratios at 12, 24 and 36 months.

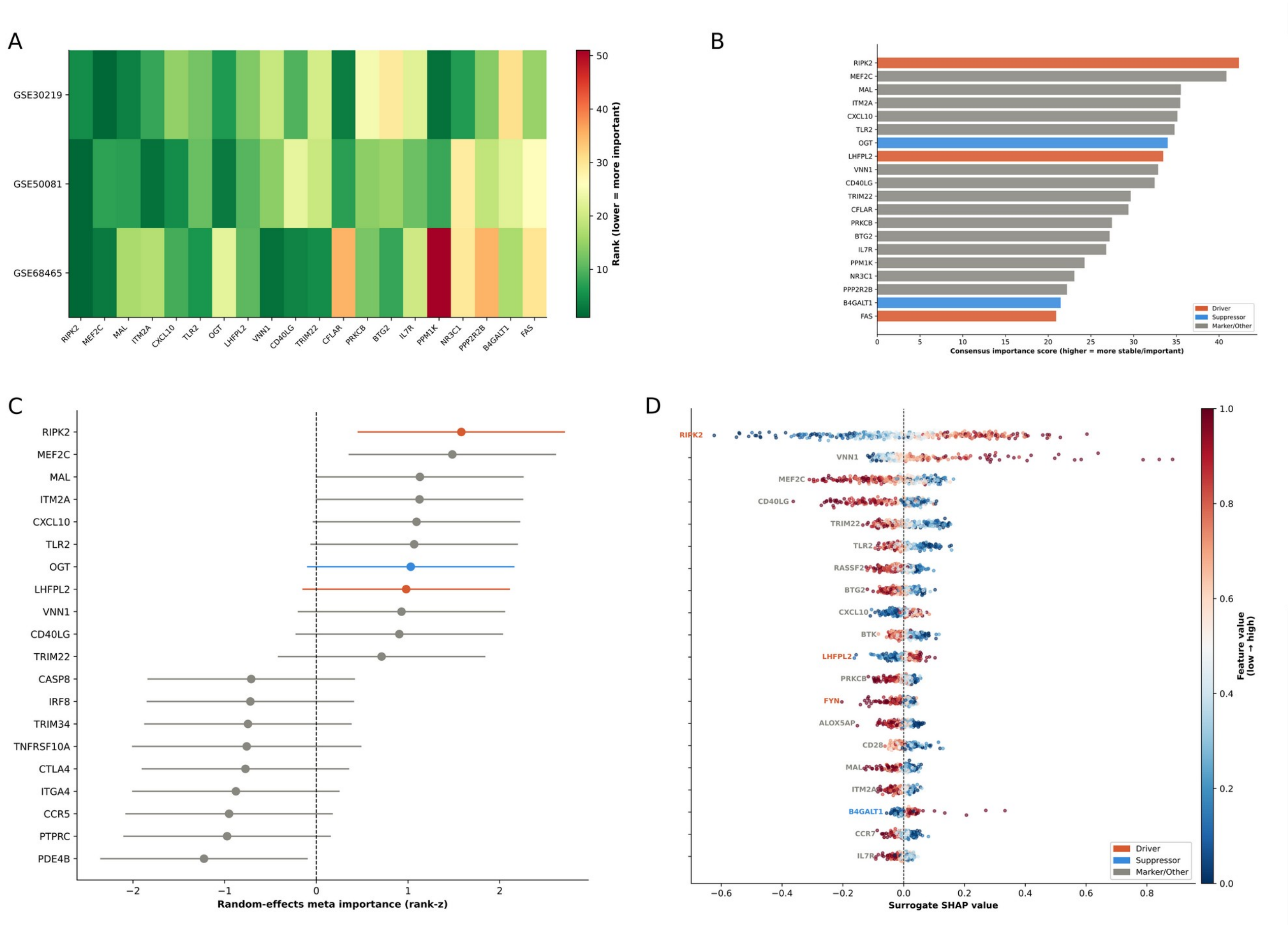

Supplementary Figure S8. Cross-cohort interpretability of the machine-learning-prioritised ferroptosis-related panel. **(A)** Feature-stability heatmap. **(B)** Consensus SHAP/permutation support. **(C)** Random-effects meta-analysis of rank-normalised feature importance. **(D)** Representative surrogate-SHAP beeswarm in GSE68465. SHAP values explain the surrogate risk-score function and are not causal gene effects.

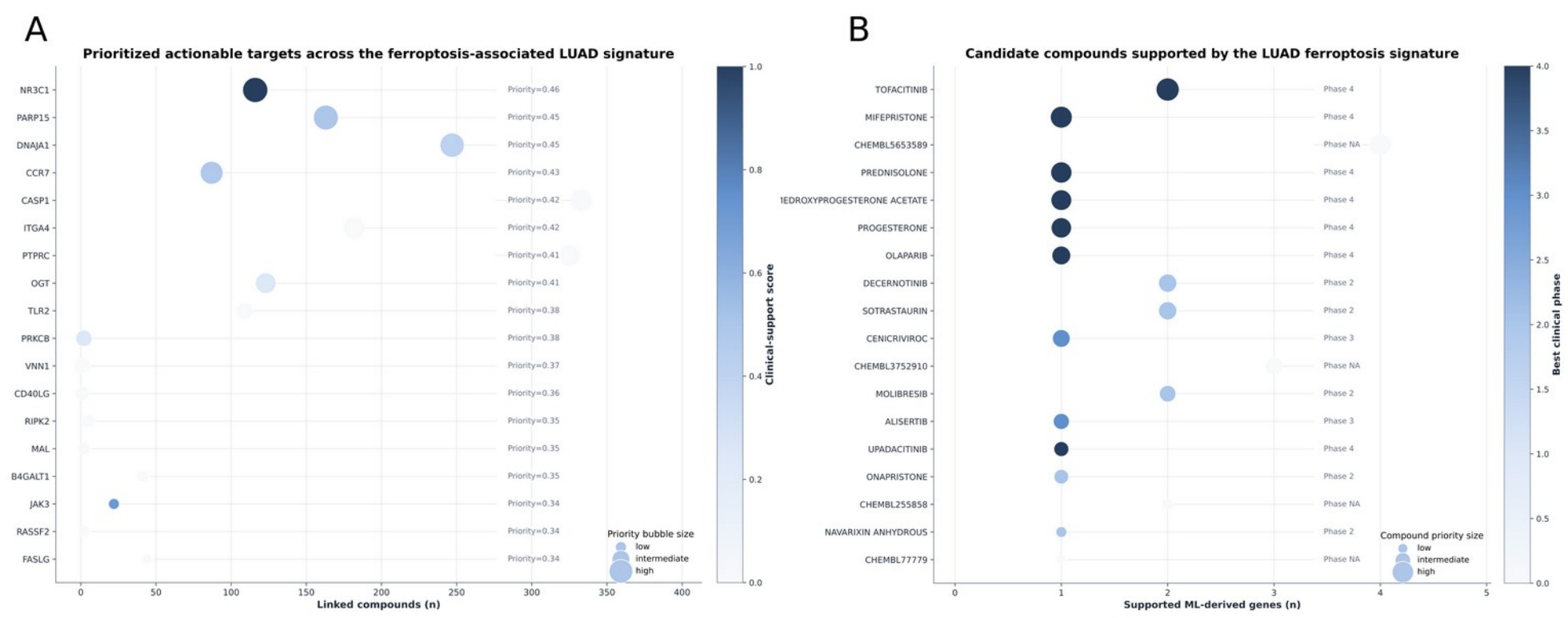

Supplementary Figure S9. Druggability and signature-reversal prioritisation. **(A)** Prioritised actionable targets; the x-axis shows the number of linked compounds, bubble size denotes target-priority category and colour denotes the clinical-support score. **(B)** Candidate compounds; the x-axis shows the number of supporting ML-derived genes, bubble size denotes compound-priority category and colour denotes the best reported clinical phase. These annotation-derived links do not imply therapeutic suitability in LUAD.

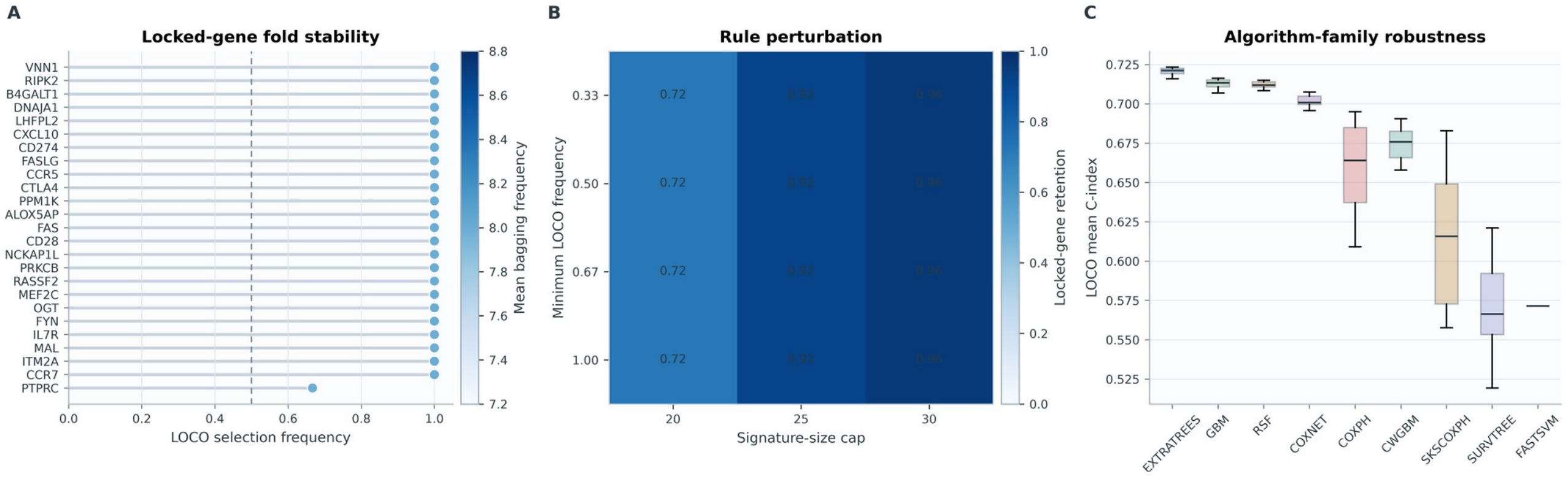

Supplementary Figure S10. Locked-signature and algorithm-family robustness. **(A)** LOCO selection frequency of locked genes. **(B)** Fraction retained under alternative LOCO-frequency thresholds and signature-size caps. **(C)** Stored LOCO mean C-indices across survival-learning families.

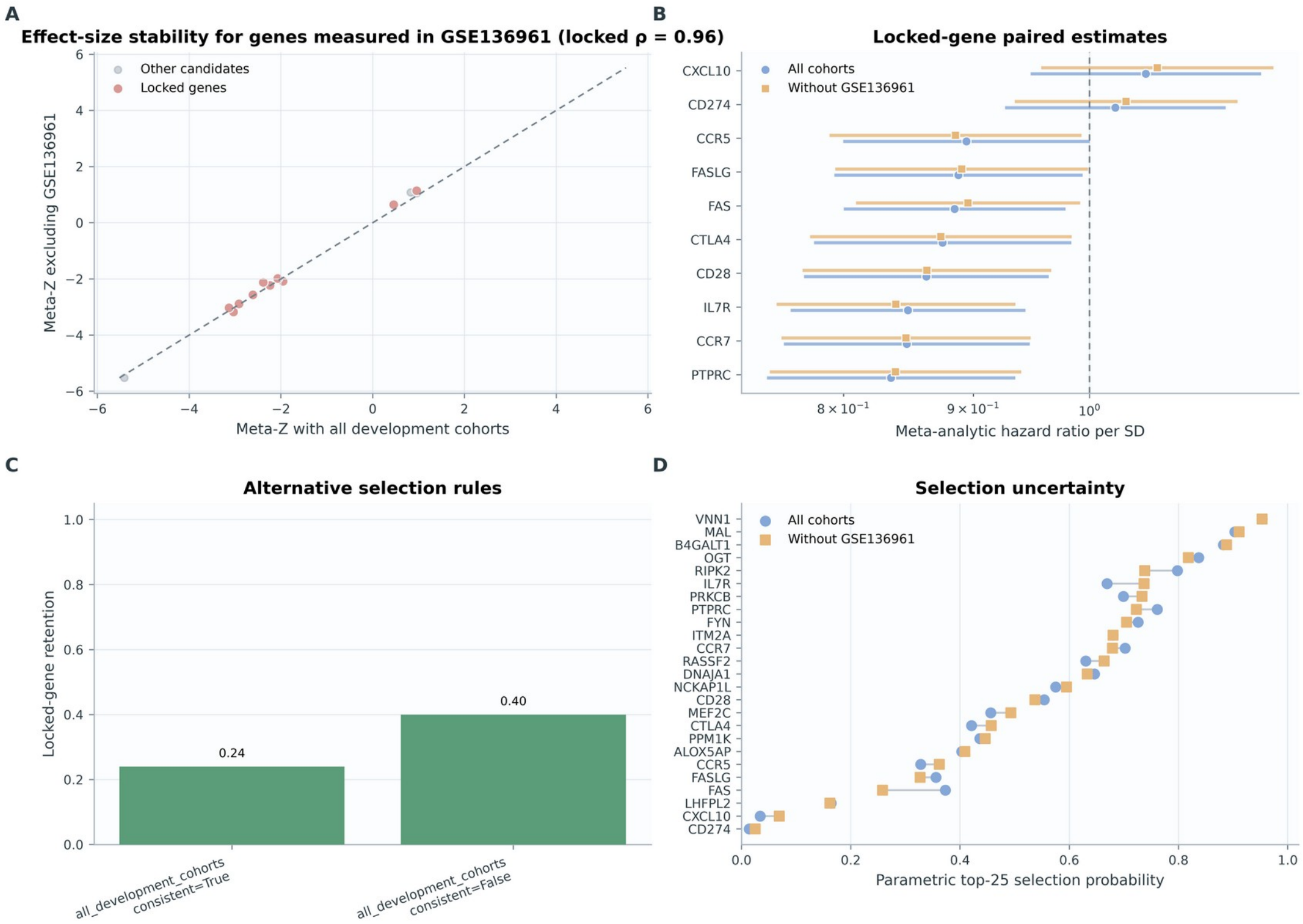

Supplementary Figure S11. Small-cohort exclusion and parameter sensitivity. **(A)** Candidate-gene meta-Z estimates with and without GSE136961. **(B)** Paired estimates for measurable locked genes. **(C)** Locked-gene retention under alternative rules. **(D)** Parametric top-25 selection probabilities. Genes absent from the platform were not imputed.

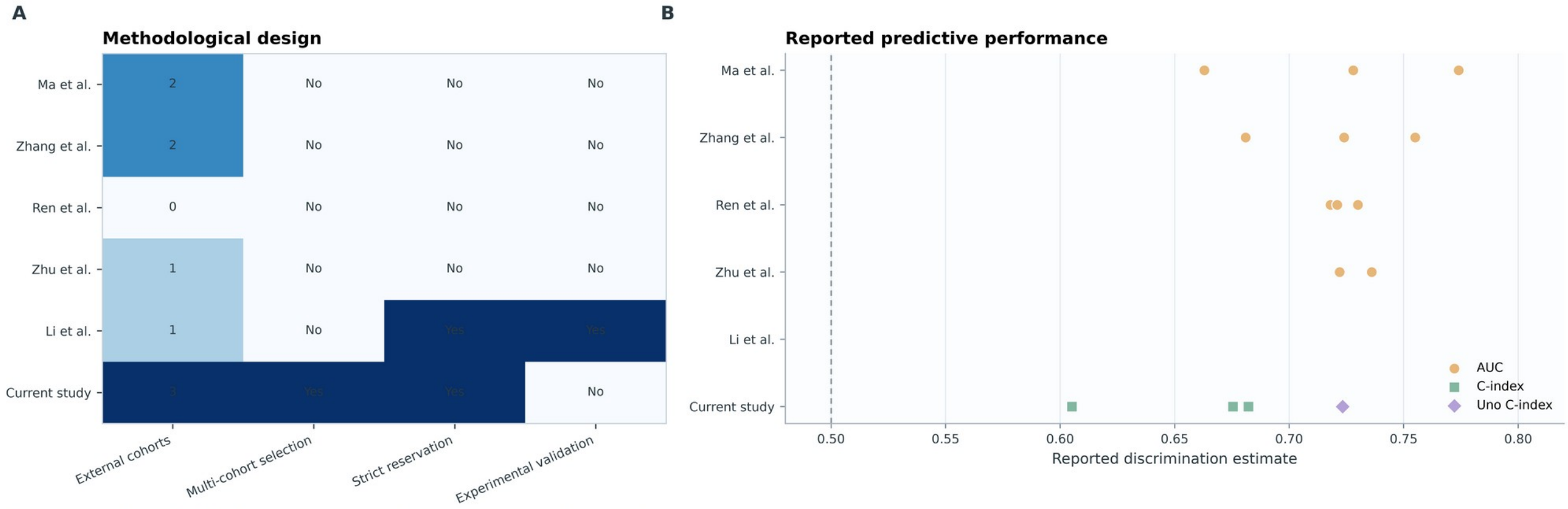

Supplementary Figure S12. Descriptive comparison with published LUAD ferroptosis-related prognostic signatures. **(A)** Methodological design matrix comparing cohort structure, multi-cohort selection, strict external reservation and experimental validation. **(B)** Reported discrimination estimates. Values are descriptive because endpoints, horizons, datasets and validation designs differ.

Supplementary Tables

Supplementary Table S1. Model terminology

**Term**	**Definition**
Locked gene panel	The fixed 25-gene set obtained by aggregating fold-level selection frequencies before external evaluation.
Development-selected reference model	The highest-ranked survival-learning configuration selected using development-only LOCO performance, then applied without cohort-specific model selection in the fixed-configuration sensitivity analysis.
Out-of-fold prediction	A development-cohort prediction produced by a model that did not use that cohort for fitting or tuning.
Stacked model	An ensemble that combines base-model out-of-fold risk scores through a second-stage survival model.
Refitted clinical model	A post hoc model used for stage-adjusted association or fixed-horizon absolute-risk estimation; it is distinct from the locked ML predictor.
Fixed-horizon model	An IPCW-weighted logistic model fitted separately at 3 and 5 years for calibration, DCA and nomogram construction when proportional hazards are not uniformly supported.
External validation	Evaluation in cohorts excluded from feature selection, tuning, ensemble optimisation and threshold selection.

Supplementary Table S2. Abbreviations used in the manuscript and Supplementary Material

**Abbreviation**	**Definition**
AJCC	American Joint Committee on Cancer
AUC	area under the time-dependent receiver operating characteristic curve
BH	Benjamini-Hochberg
CI	confidence interval
C-index	concordance index
COXNET	elastic-net penalised Cox proportional-hazards model
CoxPH	Cox proportional-hazards model
CWGBM	component-wise gradient boosting machine
DCA	decision-curve analysis
DEG	differentially expressed gene
E/O	expected-to-observed event ratio
ETX	extra survival trees
FASTSVM	FastSurvivalSVM
FDR	false-discovery rate
FRG	ferroptosis-related gene
FRGS	ferroptosis-related gene signature
GBM	gradient boosting survival analysis
GDC	Genomic Data Commons
GEO	Gene Expression Omnibus
GSEA	gene set enrichment analysis
GSVA	gene set variation analysis
HGNC	HUGO Gene Nomenclature Committee
HR	hazard ratio
ICI	immune-checkpoint inhibitor
IDI	integrated discrimination improvement
IFN	interferon
IPCW	inverse probability of censoring weighting
LASSO	least absolute shrinkage and selection operator
LOCO	leave-one-cohort-out
LUAD	lung adenocarcinoma
LUSC	lung squamous cell carcinoma
ML	machine learning
NRI	net reclassification improvement

Abbreviation	Definition
NSCLC	non-small-cell lung cancer
OOF	out-of-fold
ORA	over-representation analysis
OS	overall survival
PDB	Protein Data Bank
PH	proportional hazards
PPI	protein-protein interaction
qPCR	quantitative polymerase chain reaction
RMST	restricted mean survival time
RNA-seq	RNA sequencing
ROC	receiver operating characteristic
RSF	random survival forest
SHAP	SHapley Additive exPlanations
SKSCOXPH	scikit-survival Cox proportional-hazards model
ssGSEA	single-sample gene set enrichment analysis
STRING	Search Tool for the Retrieval of Interacting Genes/Proteins
TCGA	The Cancer Genome Atlas
TME	tumour microenvironment
TNM	tumour-node-metastasis
TPM	transcripts per million
WGCNA	weighted gene co-expression network analysis

Supplementary Table S3. Fixed-configuration external discrimination using the development-selected ExtraSurvivalTrees reference model. The same stored configuration was evaluated in all complete external cohorts.

Cohort	n	Events	Harrell's C	5-year Uno's C	HR per SD (95% CI)	3-year AUC	5-year AUC
GSE50081	181	75	0.605	0.605	1.29 (1.04-1.59)	0.639	0.654
GSE68465	442	236	0.675	0.678	1.70 (1.48-1.94)	0.720	0.699
GSE30219	289	196	0.682	0.696	1.72 (1.50-1.98)	0.763	0.745

Supplementary Table S4. Bootstrap optimism-corrected fixed-horizon performance and calibration.

Horizon	Model	Corrected AUC (95% CI)	Corrected Brier (95% CI)	Calibration slope (95% CI)	E/O ratio (95% CI)	Successful bootstraps
3 years	TNM stage	0.686 (0.622-0.749)	0.212 (0.195-0.237)	1.125 (0.727-1.737)	0.985 (0.845-1.121)	1000/1000
3 years	Signature	0.722 (0.661-0.788)	0.204 (0.184-0.229)	1.023 (0.742-1.458)	0.975 (0.834-1.106)	1000/1000
3 years	TNM + signature	0.715 (0.652-0.776)	0.206 (0.186-0.232)	1.060 (0.759-1.488)	0.977 (0.850-1.109)	779/1000
5 years	TNM stage	0.677 (0.606-0.752)	0.220 (0.202-0.246)	1.178 (0.581-2.257)	0.995 (0.884-1.113)	1000/1000
5 years	Signature	0.698 (0.619-0.782)	0.215 (0.196-0.242)	1.163 (0.710-1.956)	0.999 (0.888-1.114)	1000/1000
5 years	TNM + signature	0.693 (0.615-0.778)	0.218 (0.197-0.246)	0.979 (0.616-1.642)	1.000 (0.878-1.113)	608/1000

Supplementary Table S5. Incremental clinical value of adding the molecular score to TNM stage.

Horizon	ΔAUC	Brier improvement	Maximum incremental net benefit	Positive threshold range	Continuous NRI (95% CI)	IDI (95% CI)
3 years	+0.029	0.0066	0.0391	0.197-0.600	0.499 (0.256-0.724)	0.0325 (0.0152-0.0480)
5 years	+0.016	0.0019	0.0146	0.394-0.541	0.306 (-0.022-0.606)	0.0282 (0.0091-0.0455)

Supplementary Table S6. RMST and landmark sensitivity analyses in TCGA-LUAD.

Analysis	N / events	Estimate	95% CI	p value
RMST at 3 years	—	Low-high difference 200.6 days	143.1-254.8	—
RMST at 5 years	—	Low-high difference 406.3 days	286.5-521.6	—
12-month landmark	392 / 124	HR per SD 2.22	1.19-4.14	0.0126
24-month landmark	217 / 76	HR per SD 1.75	0.81-3.79	0.157
36-month landmark	131 / 45	HR per SD 0.70	0.27-1.81	0.466

Supplementary Table S7. Leading genes in the cross-cohort SHAP/permutation consensus.

Consensus rank	Gene	Direction in surrogate interpretation	Interpretive note
1	RIPK2	Risk	Highest consensus support
2	MEF2C	Protective	Cross-cohort consensus support
3	MAL	Protective	Cross-cohort consensus support
4	ITM2A	Protective	Cross-cohort consensus support
5	CXCL10	Risk	Cross-cohort consensus support
6	TLR2	Protective	Cross-cohort consensus support
7	OGT	Protective	Cross-cohort consensus support
8	LHFPL2	Risk	Cross-cohort consensus support
9	VNN1	Risk	Cross-cohort consensus support
10	CD40LG	Protective	Cross-cohort consensus support

Supplementary Table S8. Structurally tractable target shortlist for follow-up.

Gene	UniProt	Target class	PDB entries	Best resolution (Å)	Experimental methods	AlphaFold	Priority score	Tier
PARP15	Q460N3	Enzyme / catalytic	48	1.30	X-ray (ligand-bound ×48)	Yes	0.68	A
BTK	Q06187	Kinase	153	1.17	NMR + X-ray	Yes	0.66	A
OGT	O15294	Enzyme / catalytic	62	1.25	NMR + X-ray	Yes	0.61	A
FYN	P06241	Kinase	57	0.90	NMR + X-ray	Yes	0.58	A
RIPK2	O43353	Kinase	34	1.80	EM + NMR + X-ray	Yes	0.54	A
CCR7	P32248	GPCR / immune	5	2.10	EM + X-ray	Yes	0.52	A
CD40LG	P29965	Cytokine / immune	8	1.82	EM + X-ray	Yes	0.50	A
PTPRC (CD45)	P08575	Phosphatase / immune	6	2.30	EM + X-ray	Yes	0.49	B
ITGA4	P13612	Integrin / immune	7	2.20	EM + X-ray	Yes	0.47	B
TLR2	O60603	TLR / innate immune	13	1.80	NMR + X-ray	Yes	0.47	B

Supplementary Table S9. Fold-level stability of the locked 25-gene panel.

Gene	Locked rank	LOCO selection frequency	Mean bag frequency	Source
VNN1	1	1.00	8/8	FerrDb+WGCNA
RIPK2	2	1.00	8/8	FerrDb+WGCNA
B4GALT1	3	1.00	8/8	FerrDb+WGCNA
DNAJA1	4	1.00	8/8	FerrDb+WGCNA
LHFPL2	5	1.00	8/8	WGCNA
CXCL10	6	1.00	8/8	FerrDb+WGCNA
CD274	7	1.00	8/8	FerrDb+WGCNA
FASLG	8	1.00	8/8	FerrDb+WGCNA
CCR5	9	1.00	8/8	FerrDb+WGCNA
CTLA4	10	1.00	8/8	FerrDb+WGCNA
PPM1K	11	1.00	8/8	FerrDb+WGCNA
ALOX5AP	12	1.00	8/8	WGCNA
FAS	13	1.00	8/8	FerrDb+WGCNA
CD28	14	1.00	8/8	FerrDb+WGCNA
NCKAP1L	15	1.00	8/8	FerrDb+WGCNA
PRKCB	16	1.00	8/8	FerrDb+WGCNA
RASSF2	17	1.00	8/8	FerrDb+WGCNA
MEF2C	18	1.00	8/8	FerrDb+WGCNA
OGT	19	1.00	8/8	FerrDb+WGCNA
FYN	20	1.00	8/8	FerrDb+WGCNA
IL7R	21	1.00	8/8	FerrDb+WGCNA
MAL	22	1.00	8/8	FerrDb+WGCNA
ITM2A	23	1.00	8/8	WGCNA
CCR7	24	1.00	8/8	FerrDb+WGCNA
PTPRC	25	0.67	8/8	FerrDb+WGCNA

Supplementary Table S10. Summary of signature and model sensitivity analyses.

Robustness dimension	Estimate	Interpretation
Fold-level stability	24/25 genes selected in all LOCO folds; minimum frequency 0.67	High fold-level persistence
Stored-rule sensitivity	23/25 retained at the 0.33-1.00 LOCO-frequency perturbation with a 25-gene cap; 24/25 with a 30-gene cap	Robust to moderate cap and frequency changes
Independent re-selection	Locked-panel retention 24.0-56.0% at a 25-gene cap	Partial rather than complete recovery
Small-cohort platform coverage	10/25 locked genes measurable in GSE136961	Complete-panel exclusion sensitivity not estimable
Small-cohort exclusion effect	Direction concordance 100%; Spearman ρ=0.964, p=7.32e-06	Stable within the measurable subset
Algorithm benchmarking	150 configurations across 9 families	Reference ETX configuration selected using development-only LOCO performance

Supplementary Table S11. Descriptive comparison with published LUAD ferroptosis-related prognostic signatures.

Study	Population	Signature size	Discovery strategy	Model	Development data	External validation	Reported performance	Leakage control	In-house / experimental validation
Ma et al. (2021)	LUAD	10	Univariable Cox screening of literature-derived ferroptosis genes followed by cross-validated LASSO	LASSO-Cox risk score	TCGA-LUAD	GSE72094; GSE68465	AUC 0.728 in TCGA; 0.774 in GSE72094; 0.663 in GSE68465; risk score plus N classification 0.711 in GSE68465	Feature selection performed in one development cohort	No
Zhang et al. (2021)	LUAD	15	Kaplan-Meier and univariable Cox screening of FerrDb genes followed by LASSO-Cox	LASSO-Cox risk score	TCGA-LUAD	GSE72094; GSE68465	TCGA 1-/3-/5-year AUC 0.724/0.755/0.681; validation-cohort AUCs at corresponding horizons were reported as at least 0.681	Feature selection performed in one development cohort	No
Ren et al. (2021)	LUAD	15	Ferroptosis-gene prognostic screening in TCGA	Multigene Cox risk score and nomogram	TCGA-LUAD		TCGA 1-/2-/3-year AUC 0.718/0.730/0.721	Single-cohort feature selection and performance assessment	No
Zhu et al. (2021)	LUAD	10	Differential expression and survival filtering followed by LASSO-Cox	FR-DEG and clinical-factor prognostic model	TCGA-LUAD	ICGC cohort	Reported model AUC range 0.722-0.736 and external validation in ICGC	Feature selection performed in one development cohort	No
Li et al. (2025)	Lung cancer including LUAD and LUSC	23	WGCNA marker identification followed by benchmarking of 101 machine-learning combinations	Machine-learning ferroptosis-related signature	GSE4573; TCGA-LUAD; TCGA-LUSC	GSE13213	Reported prognostic discrimination beyond clinicopathologic factors	Cohort roles reported but strict nested leakage-control details were not equivalent to LOCO locking	Yes; limited qPCR and cell-line validation
Current study	LUAD	25	Independent WGCNA biology gate; multi-cohort bagged Cox screening; LOCO stability locking	25-gene panel; development-selected ExtraSurvivalTrees reference configuration; descriptive candidate-model benchmark	TCGA-LUAD, GSE31210, GSE136961, GSE72094	GSE50081, GSE68465, GSE30219	LOCO Uno C-index 0.723; per-cohort best single-model C-indices 0.611, 0.691 and 0.699; fixed-reference Harrell C-indices 0.605, 0.675 and 0.682	Fold-local gene selection and hyperparameter tuning; external cohorts excluded from feature selection and development tuning; fixed-model sensitivity reported separately	No

References

Consortium, U., 2015. UniProt: a hub for protein information. Nucleic acids research 43, D204–D212.

Freshour, S.L., Kiwala, S., Cotto, K.C., et al., 2021. Integration of the Drug–Gene Interaction Database (DGIdb 4.0) with open crowdsource efforts. Nucleic Acids Research 49, D1144–D1151.

Geurts, P., Ernst, D., Wehenkel, L., 2006. Extremely randomized trees. Machine learning 63, 3–42.

Graf, E., Schmoor, C., Sauerbrei, W., Schumacher, M., 1999. Assessment and comparison of prognostic classification schemes for survival data. Statistics in medicine 18, 2529–2545.

Li, Z., 2022. Extracting spatial effects from machine learning model using local interpretation method: An example of SHAP and XGBoost. Computers, Environment and Urban Systems 96, 101845.

Li, Z., Chen, Y., Hou, B., Mi, Y., Fu, C., Han, Z., ... & Zou, Z. (2025). Machine learning constructs a ferroptosis related signature for predicting prognosis and drug sensitivity in lung cancer. Cellular Oncology, 48(6), 1971-1986.

Ma, C., Li, F., & Luo, H. (2021). Prognostic and immune implications of a novel ferroptosis-related ten-gene signature in lung adenocarcinoma. Annals of Translational Medicine, 9(13), 1058.

Ochoa, D., Hercules, A., Carmona, M., Suveges, D., Baker, J., Malangone, C., Lopez, I., Miranda, A., Cruz-Castillo, C., Fumis, L., others, 2023. The next-generation Open Targets Platform: reimagined, redesigned, rebuilt. Nucleic acids research 51, D1353–D1359.

Ren, Z., Hu, M., Wang, Z., Ge, J., Zhou, X., Zhang, G., & Zheng, H. (2021). Ferroptosis-related genes in lung adenocarcinoma: prognostic signature and immune, drug resistance, mutation analysis. Frontiers in genetics, 12, 672904.

Subramanian, A., Tamayo, P., Mootha, V.K., Mukherjee, S., Ebert, B.L., Gillette, M.A., Paulovich, A., Pomeroy, S.L., Golub, T.R., Lander, E.S., others, 2005. Gene set enrichment analysis: a knowledge-based approach for interpreting genome-wide expression profiles. Proceedings of the National Academy of Sciences 102, 15545–15550.

Varadi, M., Anyango, S., Deshpande, M., Nair, S., Natassia, C., Yordanova, G., Yuan, D., Stroe, O., Wood, G., Laydon, A., others, 2022. AlphaFold Protein Structure Database: massively expanding the structural coverage of protein-sequence space with high-accuracy models. Nucleic acids research 50, D439–D444.

Zdrazil, B., Felix, E., Hunter, F., Manners, E.J., Blackshaw, J., Corbett, S., De Veij, M., Ioannidis, H., Lopez, D.M., Mosquera, J.F., others, 2024. The ChEMBL Database in 2023: a drug discovery platform spanning multiple bioactivity data types and time periods. Nucleic acids research 52, D1180–D1192.

Zhang, A., Yang, J., Ma, C., Li, F., & Luo, H. (2021). Development and validation of a robust ferroptosis-related prognostic signature in lung adenocarcinoma. Frontiers in Cell and Developmental Biology, 9, 616271.

Zhou, N., Peng, L., Luo, Q., Yin, T., Sun, H., Zhang, Y., Shi, X., Peng, X., Bao, J., Ning, Y., others, 2026. FerrDb V3: expanding the manually curated resource for regulators and disease associations from ferroptosis to regulated cell death. Nucleic Acids Research 54, D572–D582.

Zhu, G., Huang, H., Xu, S., Shi, R., Gao, Z., Lei, X., ... & Xu, S. (2021). Prognostic value of ferroptosis-related genes in patients with lung adenocarcinoma. Thoracic cancer, 12(12), 1890-1899.
